# Hybrid handcrafted and deep feature fusion for automated acute myeloid leukemia classification using TCMA-Net on a class-balanced dataset

**DOI:** 10.3389/fonc.2026.1774702

**Published:** 2026-05-14

**Authors:** Osama M. Alshehri, Zohaib Mushtaq, Muhammad Taha, Maham Ijaz, Muhammad Irfan, Malik A. Altayar, Mohammed M. Jalal, Mohammed H. Abu-Alghayth, Humood Al Shmrany, Elhashimi Eltayb Hassan

**Affiliations:** 1 Department of Clinical Laboratory Sciences, College of Applied Medical Sciences, Najran University, Najran, Saudi Arabia; 2 Department of Electrical, Electronics and Computer Systems, University of Sargodha, Sargodha, Pakistan; 3 Department of Electronics Engineering, Islamia University of Bahawalpur, Bahawalpur, Pakistan; 4 Department of Food Science and Technology, Islamia University of Bahawalpur, Bahawalpur, Pakistan; 5 Electrical Engineering Department, College of Engineering, Najran University, Najran, Saudi Arabia; 6 Department of Medical Laboratory Technology, Faculty of Applied Medical Sciences, University of Tabuk, Tabuk, Saudi Arabia; 7 Department of Medical Laboratory Sciences, College of Applied Medical Sciences, University of Bisha, Bisha, Saudi Arabia; 8 Department of Medical Laboratory, College of Applied Medical Sciences, Prince Sattam bin Abdulaziz University, Alkharj, Saudi Arabia; 9 Department of Clinical Laboratory Science, College of Applied Medical Sciences, Najran University, Najran, Saudi Arabia

**Keywords:** convolutional neural network, DenseNet201, HADNet, handcrafted features, MobileNetV2, TCMA-Net, vision transformer, acute myeloid leukemia

## Abstract

Acute myeloid leukemia (AML) is a life-threatening hematological malignancy that requires accurate and timely diagnosis for effective clinical management. However, conventional cytomorphological analysis is time-consuming, subjective, and highly dependent on expert interpretation. Existing artificial intelligence-based approaches are often limited by severe class imbalance and inadequate feature representation, which restrict their generalization capability. To address these challenges, this study proposes a robust and high-performance AML classification framework based on hybrid feature fusion and deep learning. The proposed approach integrates handcrafted features with deep representations extracted from DenseNet201 and MobileNetV2 to form the Hybrid AML Descriptor Network (HADNet), which is further combined with the Transformer-based Tri block Convolutional Multi head Attention Network (TCMA-Net) classifier to capture both local and global contextual information. In addition, a Synthetic Acquisition Artifact Augmentation (S3A) strategy is introduced to mitigate class imbalance by generating realistic variations in illumination and noise. Extensive experiments were conducted on the AML cytomorphology Ludwig Maximilian University (LMU) dataset, where the proposed framework achieved a testing accuracy of 99.20%, outperforming traditional machine learning models and deep learning baselines. The effectiveness of the approach is further validated through ablation studies and external evaluation on the acute lymphoblastic leukemia (ALL) dataset, demonstrating strong robustness and generalization across different data distributions. Overall, the results indicate that the integration of handcrafted and deep features significantly enhances classification performance, stability, and reliability. The proposed framework provides a promising solution for automated AML diagnosis and has the potential to support clinical decision-making systems.

## 1 Introduction

Bone marrow (BM), located within the cavities of bones, is a soft yet resilient tissue responsible for hematopoiesis, the continuous production of millions of new blood cells each day to maintain normal physiological activity. Blood is composed of approximately 80% water and 20% solids and contains erythrocytes [red blood cells (RBCs)], leukocytes [white blood cells (WBCs)], thrombocytes, and plasma. Among these components, white blood cells constitute approximately 1% of the total blood content, corresponding to roughly one leukocyte for 100 red blood cells (1). When the BM produces abnormal or immature leukocytes, the condition is referred to as leukemia, a malignant neoplasm affecting blood-forming tissues (2). Leukemia represents a serious and potentially life-threatening disease that requires early diagnosis and treatment to prevent fatal outcomes. Leukemia can be broadly categorized into four major types: acute lymphoblastic leukemia (ALL) (3), acute myeloid leukemia (AML) (4), chronic lymphocytic leukemia (CLL) (5), and chronic myeloid leukemia (CML) (6). Among these, AML is considered one of the most aggressive forms and is associated with a high mortality rate. It is estimated to cause approximately 11,000 deaths annually in the United States, with a 5-year survival rate of only 28.7%. AML can arise due to several factors, including somatic DNA mutations, inherited genetic predisposition, or germline mutations inherited from parents (7). The disease weakens the immune system by causing uncontrolled production of immature white blood cells (8). Leukemic cells are also capable of evading immune surveillance through multiple mechanisms, such as deletion of major histocompatibility complex (MHC) antigens, expression of inhibitory ligands, inhibition of activating receptors, disruption of surface ligand interactions, and regulation of soluble factors within the tumor microenvironment (9). Several environmental and lifestyle factors have been associated with AML development. Tobacco use is considered one of the most significant risk factors, while exposure to carcinogenic chemicals such as benzene and formaldehyde may also contribute to disease development (10). Current treatment strategies for AML include cytotoxic chemotherapy, allogeneic stem-cell transplantation, and modern targeted therapies that have expanded the clinical treatment landscape (11).

Despite the availability of these treatments, accurate and timely diagnosis of AML remains a significant challenge for clinicians and researchers. Diagnostic procedures often require specialized laboratory tests and expert microscopic examination of blood or BM samples. These procedures are time-consuming and expensive and may not be readily available in many healthcare settings. In addition, limited access to advanced diagnostic equipment, insufficient clinical training, and a lack of diagnostic resources can further complicate early detection of the disease. Inaccurate or delayed diagnosis may lead to over-treatment or under-treatment, negatively affecting patient outcomes (12). Traditional manual examination of blood smear images by hematologists is also subject to inter-observer variability and may lack precision in complex cases. Consequently, there is a growing need for automated diagnostic systems capable of assisting clinicians in detecting leukemia more efficiently and reliably. Recent advances in artificial intelligence (AI), particularly machine learning (ML) and deep learning (DL), have demonstrated significant potential in medical image analysis and disease diagnosis. DL models have been widely applied to analyze microscopic blood cell images, enabling automated leukemia detection with improved speed and accuracy. These systems are particularly beneficial in resource-limited clinical environments where access to specialized expertise may be limited. Moreover, ML approaches applied to genomic and cytomorphological data have facilitated the identification of previously unknown AML subtypes, thereby creating new opportunities for improved diagnostic strategies. However, existing approaches still face several challenges, including limited utilization of complementary feature representations, difficulties in handling class imbalance in medical datasets, and insufficient modeling of both local morphological patterns and global contextual relationships in cell images.

To address these challenges, this study proposes an AI framework for automated AML classification that integrates DL and Transformer-based techniques for improved diagnostic performance. The proposed approach combines advanced feature extraction strategies with an effective classification architecture to enhance the accuracy and robustness of AML detection from microscopic blood cell images. Additionally, to assess the robustness and generalization capability of the proposed framework, external validation is performed on an independent dataset (ALL) obtained from a different source. The main contributions of this work are summarized as follows:

- introduced a novel feature fusion model, Hybrid AML Descriptor Network (HADNet), that integrates handcrafted and deep features to improve AML classification performance;
- proposed the Synthetic Acquisition Artifact Augmentation (S3A) data balancing method to mitigate class imbalance in the AML dataset; and
- utilized the Tri block Convolutional Multi head Attention Network (TCMA-Net) classifier, which effectively combines a convolutional neural network (CNN) and Vision Transformer (ViT) for improved classification accuracy.

The paper has the following structure of description: Section 2 reviews the previously available related studies, Section 3 presents the experimental content and methodology, Section 4 describes the experimental results and compares them with benchmark models, and Section 5 concludes the research and outlines the future directions.

## 2 Related study

AI has been widely applied in medical image analysis for automated WBC classification; however, accurate detection of leukemic cells remains challenging due to complex and atypical cell morphology. Existing approaches for leukemia detection can generally be categorized into traditional ML methods, DL models, and hybrid feature fusion techniques. A comparative summary of representative studies is provided in **Table 1**.

**Table 1:** Summary of representative AML classification studies highlighting their contributions and limitations.

Authors	Contributions	Limitations
Das et al. (13)	Proposed a hybrid Random Forest–Support Vector Machine (RF–SVM) leukemia classification framework integrating AWOLSE feature selection and watershed segmentation for automated detection of acute lymphoblastic leukemia (ALL) and acute myeloid leukemia (AML).	Small datasets (108 ALL and 80 AML images) limiting generalization
Ramya and Lakshmi (14)	Developed a Fractional Black Widow-based Neural Network (FBW-NN) combined with Adaptive Fuzzy Entropy (AFE) for leukemia cell segmentation and classification.	Additive noise analysis and generalization processing for image de-noising
Yadav et al. (15)	Introduced the 3SNet deep convolutional neural network (CNN) architecture incorporating multi-scale feature fusion with handcrafted HOG and LBP descriptors for AML classification.	Requirement for three-scale image input for training and reduction of computation cost of model
Lu et al. (16)	Proposed MAE4AL, an active learning framework based on Masked Autoencoder (MAE) representations for blood cell classification.	Requiring broader validation and attention to class imbalance
Kasim et al. (17)	Developed a multiclass leukemia classification approach combining pre-trained CNN models with machine learning classifiers (InceptionV3 + SVM).	Lack of dataset size, leading to overfitting and reduced generalization

### 2.1 Machine learning-based leukemia classification

Early research on leukemia classification mainly relied on morphological and texture features combined with traditional ML algorithms. Das et al. (13) proposed a hybrid Random Forest (RF)–Support Vector Machine (SVM) framework integrating Adaptive Weight-Optimized Level Set Evolution (AWOLSE) and watershed segmentation, achieving 99.07% accuracy for ALL and 96.25% for AML detection. However, the study relied on relatively small datasets consisting of only 108 and 80 images for ALL and AML, respectively, which limits its generalization capability. Jagadev and Virani (18) proposed a multiclass SVM framework utilizing K-means clustering, watershed segmentation, and Hue, Saturation, Value (HSV) color space analysis for leukemia detection. Although the approach demonstrated promising classification ability, the work was limited by a dataset of only 220 images and lacked the capability to classify fine-grained leukemia subtypes. Similarly, Liu and Hu (19) introduced a Random Forest-based classification framework combined with a Broad Learning System (BLS) that utilized fused morphological and radiomics features to differentiate AML-M1 and AML-M2 subtypes. Their model achieved an accuracy of 99.8%, but the study relied on only 50 BM images and therefore requires further validation on larger datasets. In another study focusing on AML detection, Dasariraju et al. (20) extracted 16 handcrafted features (HCFs) from leukocyte images, including nucleus color descriptors, and applied a Random Forest classifier. Their approach achieved a detection rate of 92.99% and a classification rate of 93.45%. Despite these encouraging results, traditional ML approaches depend heavily on manual feature extraction, which may limit their ability to capture complex morphological variations present in leukemic cells.

### 2.2 Deep learning-based leukemia classification

With the advancement of DL, CNNs have become widely adopted for automated leukemia classification due to their ability to learn hierarchical features directly from image data. Elhassan et al. (21) proposed the GT-DCAE framework to address class imbalance by utilizing a two-stage DCAE-CNN architecture for classification, achieving an accuracy of 97%. Similarly, Lazouni et al. (22) introduced a modified U-Net architecture designed for segmentation tasks, achieving an accuracy of 0.9008. Further advancements were presented by Ramya and Lakshmi (14), who proposed the Fractional Black Widow-based Neural Network (FBW-NN) combined with an Adaptive Fuzzy Entropy (AFE) model for segmentation and classification tasks, achieving an overall accuracy of 96.56%. In another study, Sanusi et al. (23) utilized Random Projections combined with Multivariate Shewhart Control Charts (MSCC) to track large-scale leukemia image data belonging to Basophil (BAS) and Promyelocyte (PMO) classes. More recent DL studies have explored advanced CNN architectures. Yadav et al. (15) proposed Three-Scale Deep Convolutional Neural Network (3SNet), a deep CNN architecture incorporating multi-scale feature fusion along with handcrafted Histogram of Oriented Gradients (HOG) and Local Binary Pattern (LBP) descriptors, achieving an average accuracy of 98.16% on the AML cytomorphology LMU dataset. Additionally, Lu et al. (16) introduced Masked Autoencoder for Active Learning (MAE4AL), an active learning framework based on self-supervised Masked Autoencoder (MAE) representations, achieving an optimal accuracy of 96.47% for blood cell classification.

### 2.3 Hybrid feature fusion approaches

To further improve classification performance, several researchers have explored hybrid approaches that integrate HCF with DL representations. Elhassan et al. (24) applied Cyan, Magenta, Yellow, and Key (Black) (CMYK)-moment localization to identify the region of interest and combined it with a CNN-based feature fusion strategy, achieving 97.57% accuracy on the primary dataset. Similarly, Prasad Palli et al. (25) proposed a Dense Squeeze Network combined with Dilated Convolutional Spatial Pyramid Pooling (Dilated CSPP) to capture both multi-level dense features and multi-scale contextual information. Their model also incorporated an Improved Artificial Fish Swarm (AFS) optimization algorithm, achieving an overall classification accuracy of 99%. Other hybrid frameworks have also been explored. Kasim et al. (17) proposed a multiclass classification framework integrating pre-trained CNN models with ML classifiers, achieving a maximum accuracy of 88% using an InceptionV3 and SVM combination. However, the model suffered from overfitting due to the relatively small dataset of 390 images. Similarly, Himel et al. (26) proposed a two-stage feature fusion ensemble based on EfficientNetB7 and MobileNetV3Large architectures, achieving an accuracy of 99.3% for multiclass leukemia diagnosis. Despite its strong performance, the approach still requires further architectural refinement to ensure robustness with a less cost-efficient model under diverse real-world clinical imaging conditions.

### 2.4 Limitations of existing approaches

Despite promising results in automated leukemia classification, existing methods face several limitations, including reliance on HCF, CNN-only architectures that may overlook complementary feature representations, evaluation on small datasets, and severe class imbalance that affects model generalization. Moreover, many approaches fail to effectively capture both local morphological patterns and global contextual relationships in cell images. To address these challenges, this study proposes a hybrid feature fusion framework integrated with a CNN–Transformer-based classification model for improved AML classification.

## 3 Materials and methods

**Figure 1:**
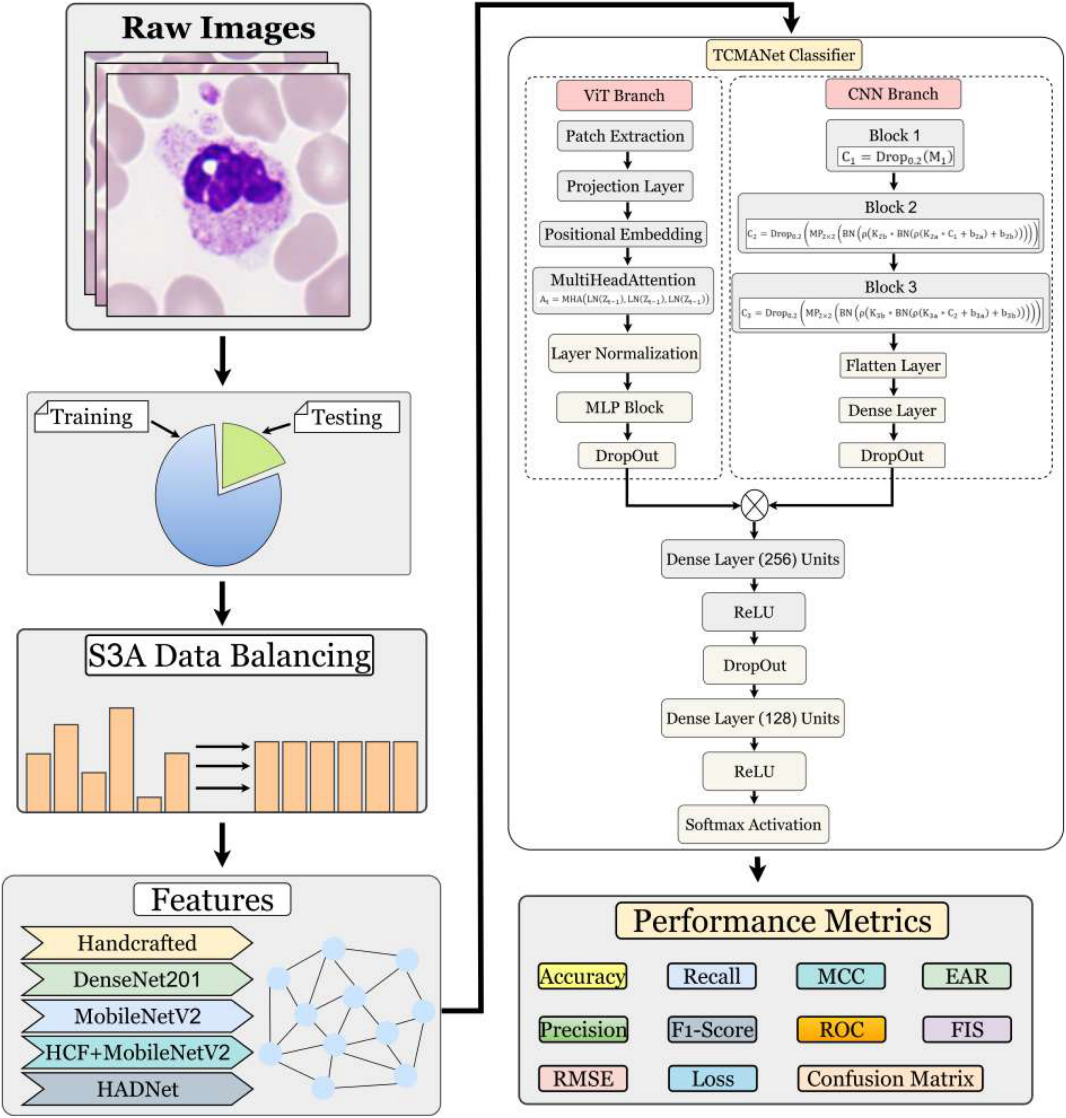
General block diagram of proposed research methodology. The diagram illustrates the workflow from raw image input, S3A data balancing, and feature extraction to the TCMA-Net Classifier involving ViT and CNN branches. S3A, Synthetic Acquisition Artifact Augmentation; TCMA-Net, Tri block Convolutional Multi head Attention Network; ViT, Vision Transformer; CNN, convolutional neural network.

In this section, data balancing, feature extraction, and classification methods will be discussed, which were applied in this research as shown in **Figure 1** for attaining higher accuracy and better results. The novel methods were designed to strengthen the model’s performance.

### 3.1 Dataset overview

**Figure 2:**
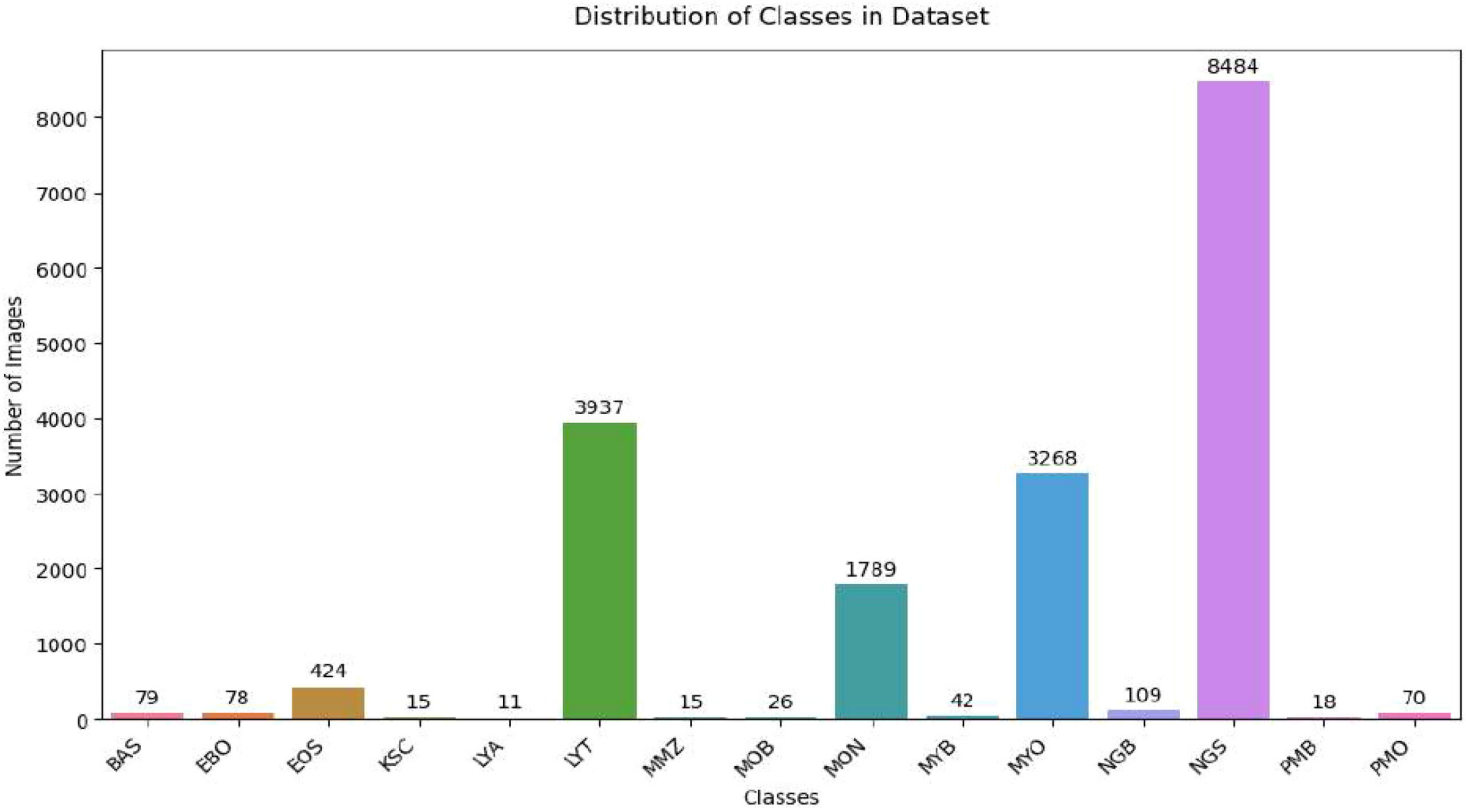
Class-wise distribution of AML cytomorphology dataset samples, showing the number of images per class and highlighting class imbalance. AML, acute myeloid leukemia.

The present study utilized the AML cytomorphology LMU dataset, which contains single-cell morphological images of leukocytes obtained from peripheral blood smears of patients diagnosed with AML. The dataset consists of 18,365 cell images collected from 100 AML patients and 100 healthy individuals (27). All samples were collected at Munich University Hospital between 2014 and 2017 using an M8 digital microscope with ×100 magnification under oil immersion. The dataset contains 15 cytomorphological classes representing different stages of leukocyte development. The complete list of classes is provided in **Table 2**, while **Figure 2** illustrates the class-wise distribution of samples. As shown in **Figure 2**, the dataset exhibits class imbalance, where certain classes contain significantly more samples than others. To ensure reliable model evaluation, the dataset was first divided into training and testing subsets using an 80:20 split. Data balancing using augmentation techniques was then applied only to the training set to address class imbalance and improve model generalization, while the test set remained unchanged to ensure unbiased evaluation. In addition to the primary dataset, an independent ALL dataset was utilized for external validation to assess the robustness and generalization capability of the proposed framework on Kaggle. The ALL dataset consists of microscopic blood smear images for leukemia classification and was obtained from a different source, enabling cross-dataset evaluation under varying data distributions.

**Table 2:** Class-wise distribution of AML cytomorphology LMU dataset.

Cell class	Number of images
Neutrophil (segmented) (NGS)	8,484
Lymphocyte (typical) (LYT)	3,937
Myeloblast (MYO)	3,268
Monocyte (MON)	1,789
Eosinophil (EOS)	424
Neutrophil (band) (NGB)	109
BAS	79
Erythroblast (EBO)	78
PMO	70
Myelocyte (MYB)	42
Monoblast (MOB)	26
Promyelocyte (bilobed) (PMB)	18
Smudge cell (KSC)	15
Metamyelocyte (MMZ)	15
Lymphocyte (atypical) (LYA)	11

AML, acute myeloid leukemia.

### 3.2 Proposed S3A data balancing method

The AML image dataset herein has a noticeably high level of class imbalance, which may bias themodel training process and limit its generalization. To address this problem, S3A is developed as adata-balancing approach by uniting oversampling steps with the illumination and noise-basedtransformations (**Algorithm 1**), therefore synthetically enriching the minority samples while maintaining the spatial coherence as well (Equation 1).

(1) $$D^{\text{raw}}={\left\{\left(I_{i},l_{i}\right)\right\}}_{i=1}^{N}$$

Here, $I_{i}\in {\mathbb{R}}^{H\times W\times C}$ denotes the *i*th image with dimensions *H* × *W* and *C* channels, and $l_{i}\in \left\{1,2,\ldots ,K\right\}$ its class label. *N* and *K* denote the total number of samples and classes, respectively. Each *I_i_* is a pixel intensity matrix enabling augmentation transformations.

To balance class distribution, the target sample size *T_i_* was set to 2,000. For each class c∈{1,2,…,K}, *N_c_* represents the number of images $I_{i}\in D^{\text{raw}}$ whose label *l_i_* = *c*. Classes with *N_c_*< *T_i_* were identified by S3A for augmentation to reach the target. For an underrepresented class *c* with *N_c_*< *T_i_*, the additional images Δ*_c_* were calculated as ${\text{Δ}}_{c}=T_{i}-N_{c}$. The S3A framework then produced Δ*_c_* augmented images using a transformation set *A_F_*, which included brightness adjustment *B_F_*, contrast enhancement *C_F_*, Gaussian noise $G_{F}^{n}$, salt-and-pepper noise SP*_F_*, Gaussian blur $G_{F}^{B}$, and distortion *D_F_* (Equation 2).

(2) $$I^{F}:{\mathbb{R}}^{H\times W\times C}\to {\mathbb{R}}^{H\times W\times C}$$

The individual *I^F^* represents a specific photometric perturbation, introducing synthetic acquisition artifacts while preserving class semantics. These changes incorporate, (Equation 3)

(3) $$B_{F}\left(i\right)=\alpha \cdot i$$

The illumination factor, represented by *α*, was sampled on a uniform distribution bounded by 0.85 to 1.12 to emulate the stochastic variability in lighting situations.

(4) $$C_{F}\left(i\right)=\beta \cdot \left(i-\mu \right)+\mu$$

*β* was drawn from a uniform distribution (Equation 4) within [0.85, 1.2], and *µ* is the mean pixel intensity of the image. This adjusts pixel intensities relative to *µ* and boosts intra-class diversity.

(5) $$G_{F}^{n}\left(i\right)=i+\gamma ,\;\gamma ∼N\left(0,\sigma^{2}\right),\;\sigma =5$$

where *γ* introduces zero-mean Gaussian noise (Equation 5), simulating minor acquisition artifacts.

(6) $$SP_{F}\left(i\right)=\{\begin{array}{ll} 0, & \text{with probability }\rho \\ 255, & \text{with probability }\rho =0.002\\ i, & \text{otherwise} \end{array}$$

This creates limited high- and low-intensity pixel (Equation 6) disruptions copying sensor noise.

(7) $$G_{F}\left(i\right)=GB\left(i,c=\left(3,3\right)\right)$$

Here, *GB* represents the Gaussian blur (Equation 7).

Slight blurring emulates defocusing or motion artifacts commonly found in microscopy.

(8) $$D_{F}\left(i\right)=\{\begin{array}{ll} 0, & \text{if }\omega <P^{d}\\ i, & \text{otherwise} \end{array}$$

The *ω* was sampled from a uniform distribution between 0 and 1, while the dropout probability *P^d^* at 0.005 was fixed (Equation 8).

Random pixel suppression enhances robustness to partial information loss. The final augmented image was obtained through the composition of multiple transformations (Equation 9),

(9) $$I_{i}^{'}=F_{i_{m}},F_{i_{m-1}},\ldots ,F_{i_{m-n}}(I_{i}))$$

Each $f_{i_{j}}\in F$ is a randomly selected function. This stochastic composition ensures diverse synthetic artifacts while retaining semantic consistency. The transformation set was *F* = *A_F_*. For each class *c*, the augmented dataset $D^{{\text{raw}}^{\prime }}$ was formed such that if $N_{c}\geq T_{i}$, only the original images ${\left\{I_{i}\right\}}_{i=1}^{T_{i}}$ (Equation 10) were retained. If $N_{c}<T_{i}$, the dataset was formed by the union of original images ${\left\{I_{i}\right\}}_{i=1}^{N_{c}}$ and the augmented images ${\left\{{I_{j}}^{\prime }\right\}}_{j=1}^{{\text{Δ}}_{c}}$, where ${\text{Δ}}_{c}=T_{i}-N_{c}$. The complete balanced dataset across all classes is

(10) $$D^{{\text{raw}}^{\prime }}=\overset{K}{\underset{c=1}{\cup }}D_{c}^{{\text{raw}}^{\prime }}$$

ensuring that $\left|D_{c}^{{\text{raw}}^{\prime }}\right|=T_{i}$ for all *K*, thus rectifying class imbalance while embedding synthetic acquisition artifacts.

### 3.3 Handcrafted features

HCFs represent a set of manually designed image descriptors that capture meaningful structural information from microscopic images. These descriptors are widely used in classification and analysis tasks and typically include shape, color, texture, and fractal features, each describing different aspects of the visual characteristics of the object. Prior to extracting morphological descriptors, leukocyte regions were automatically segmented to obtain clean object masks for reliable geometric analysis. The segmentation masks were generated using Otsu thresholding followed by connected component labeling to isolate the largest region corresponding to the leukocyte. This segmentation step ensures that the extracted descriptors accurately represent the cell structure while minimizing background interference.

Among handcrafted descriptors, shape features play an important role in describing the morphological characteristics of leukocytes. Initially, a total of eight shape descriptors were extracted from the segmented cell regions using the *regionprops* function from the *scikit-image* library. To identify the most informative attributes, a feature importance analysis using an RF model was applied to the extracted descriptors, and the same selection strategy was subsequently used for other HCF groups. Based on the obtained importance scores, the four most discriminative shape descriptors were selected, including *area*, *major axis length*, *minor axis length*, and *solidity*. The area $S_{A}^{f}$ represents the total number of object pixels and is defined as the summation of pixels *x_i_*, where *x_i_* = 1 for object pixels and *x_i_* = 0 for background pixels across the total number of pixels *N*. The major axis length *l*_1_ corresponds to the longest axis of the ellipse that best fits the segmented leukocyte, while the minor axis length *l*_2_ represents the length of the perpendicular shorter axis of the same ellipse. These descriptors capture the size and elongation characteristics of the cell structure. The solidity $S_{s}^{f}$ measures the compactness of the leukocyte and is defined as the ratio between the object area $S_{A}^{f}$ and the convex hull area $S_{A_{convex}}^{f}$. The selected descriptors were combined to form the shape feature vector (Equation 11):

Algorithm 1 **Balanced augmented dataset generation.**

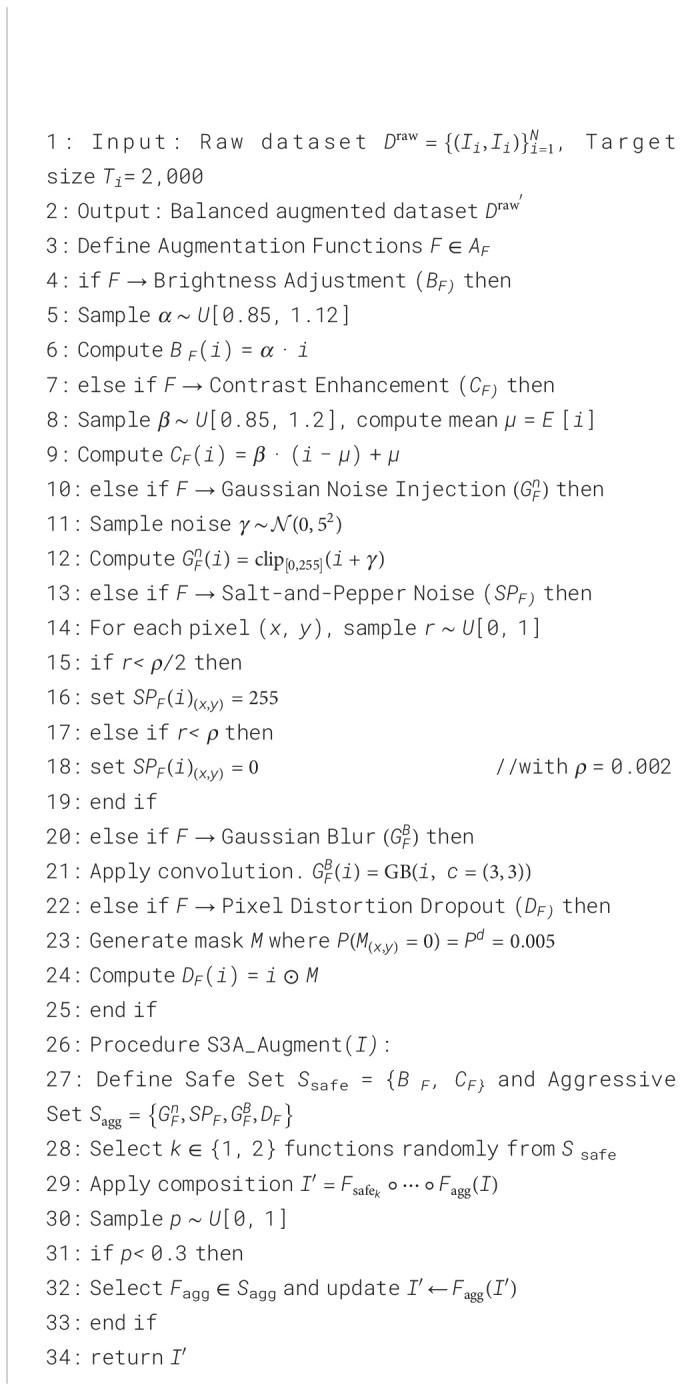

(11) $$F_{shape}={\left[S_{A}^{f},\;l_{1},\;l_{2},\;S_{s}^{f}\right]}^{T}$$

In addition to morphological descriptors, color features were extracted to capture the chromatic characteristics of leukocytes. Color information provides important cues about staining patterns and cellular composition that assist in distinguishing between different leukocyte types. Initially, 12 color descriptors were extracted from the RGB, HSV, and Lab color spaces. Using the same feature importance strategy described earlier, the six most informative color descriptors were selected for further analysis. These include the *standard deviation of the red channel*, *mean hue*, *standard deviation of hue*, *mean saturation*, *standard deviation of saturation*, and *mean Lab-B channel*. The standard deviation of the red channel $C_{R}^{\sigma }$ represents the variation of red intensity values *R_i_* around their mean $C_{R}^{\mu }$. The mean hue $C_{H}^{\mu }$ represents the average value of hue pixels *H_i_* in the HSV color space. The standard deviation of hue $C_{H}^{a}$ measures the dispersion of hue values around the mean hue $C_{H}^{\mu }$. Similarly, the mean saturation $C_{S}^{\mu }$ represents the average saturation values *S_i_*, indicating color intensity, while the standard deviation of saturation $C_{S}^{\sigma }$ captures the variations of saturation values around the mean $C_{S}^{\mu }$. In addition, the mean Lab-B channel $C_{B}^{\mu }$ represents the average value of the *B_i_* component in the Lab color space, reflecting the balance between blue and yellow color components. The selected color descriptors were concatenated to form the color feature vector (Equation 12):

(12) $$F_{colour}={\left[C_{R}^{\sigma },\;C_{H}^{\mu },\;C_{H}^{\sigma },\;C_{S}^{\mu },\;C_{S}^{\sigma },\;C_{B}^{\mu }\right]}^{T}$$

To further describe structural patterns within leukocyte images, texture features were extracted to characterize the spatial distribution of pixel intensities. Initially, 13 texture descriptors were computed using intensity statistics and Gray-Level Co-occurrence Matrix (GLCM) representations. Applying the same feature importance strategy, the seven most discriminative texture descriptors were selected. These include *standard deviation*, *skewness*, *kurtosis*, *entropy*, *GLCM contrast*, *GLCM correlation*, and *GLCM homogeneity*. The standard deviation $T_{\sigma }^{f}$ measures the spread of pixel intensities *x_i_* around the mean intensity $T_{\mu }^{f}$, representing the contrast level of the texture. The skewness $T_{s}^{f}$ quantifies the asymmetry of the intensity distribution, while the kurtosis $T_{k}^{f}$ measures the peakedness of the distribution. The entropy $T_{en}^{f}$ represents the randomness of pixel intensities based on the probability *p_i_* of intensity values. Additionally, GLCM contrast $G_{contrast}^{T}$ measures intensity differences between neighboring pixels, GLCM correlation $G_{corr}^{T}$ evaluates linear dependencies between pixel pairs, and GLCM homogeneity $G_{hom}^{T}$ measures the closeness of elements in the co-occurrence matrix to its diagonal. These descriptors were combined to form the texture feature vector (Equation 13):

(13) $$F_{texture}={\left[T_{\sigma }^{f},\;T_{s}^{f},\;T_{k}^{f},\;T_{en}^{f},\;G_{contrast}^{T},\;G_{corr}^{T},\;G_{hom}^{T}\right]}^{T}$$

Finally, fractal features were extracted to describe the self-similar and scale-invariant characteristics of leukocyte structures. These descriptors provide a quantitative measure of geometric complexity and boundary irregularity across different spatial scales. In this study, two fractal descriptors were extracted using the box-counting method, namely, *fractal dimension* and *lacunarity*. The fractal dimension $F_{D}^{f}$ measures structural complexity and is defined as Equation 14

(14) $$F_{D}^{f}=-\frac{\log\;N_{b}}{\log\;b}$$

where *N_b_* represents the number of boxes required to cover the object and *b* is the box size. The lacunarity $F_{L}^{f}$ measures the heterogeneity or gap distribution of the structure and is defined as Equation 15

(15) $$F_{L}^{f}=\frac{\text{variance of box count}}{{\left(\text{mean of box count}\right)}^{2}}$$

These descriptors were combined to form the fractal feature vector (Equation 16):

(16) $$F_{fractal}={\left[F_{D}^{f},\;F_{L}^{f}\right]}^{T}$$

After extracting the most informative descriptors from each HCF category, the selected features from the shape, color, texture, and fractal groups were concatenated to construct the final handcrafted representation. This feature fusion combines the complementary morphological, chromatic, structural, and geometric characteristics of leukocytes into a unified representation. In total, 19 HCF were obtained after fusion, consisting of four shape features, six color features, seven texture features, and two fractal features. The final fused feature vector is expressed as

(17) $$F_{fused}=[F_{shape}\;∥\;F_{colour}\;∥\;F_{texture}\;∥\;F_{fractal}]$$

**Figure 3:**
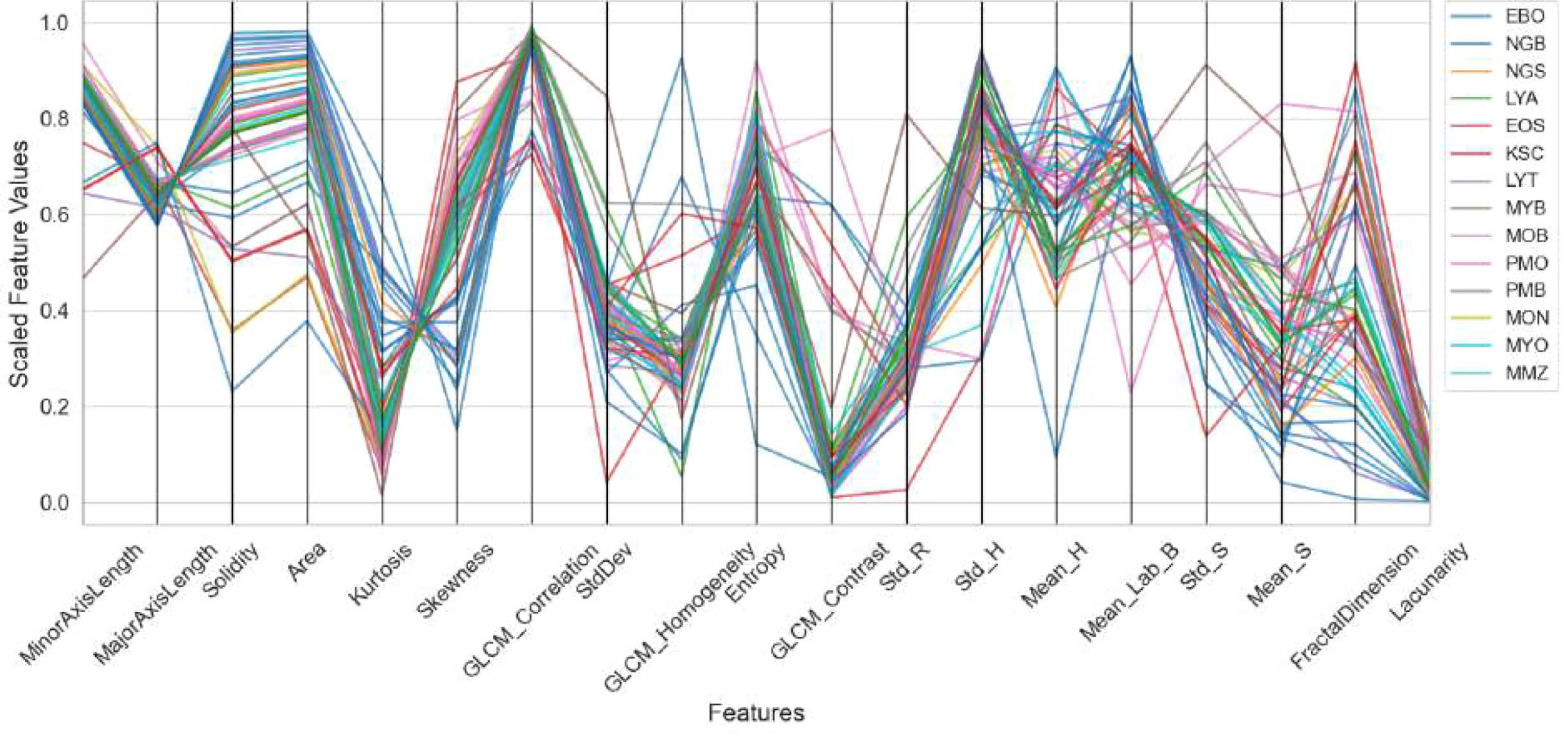
Parallel coordinates plot of HCF, illustrating feature-wise variation across samples and highlighting inter-class separability. HCF, handcrafted feature.

**Figure 4:**
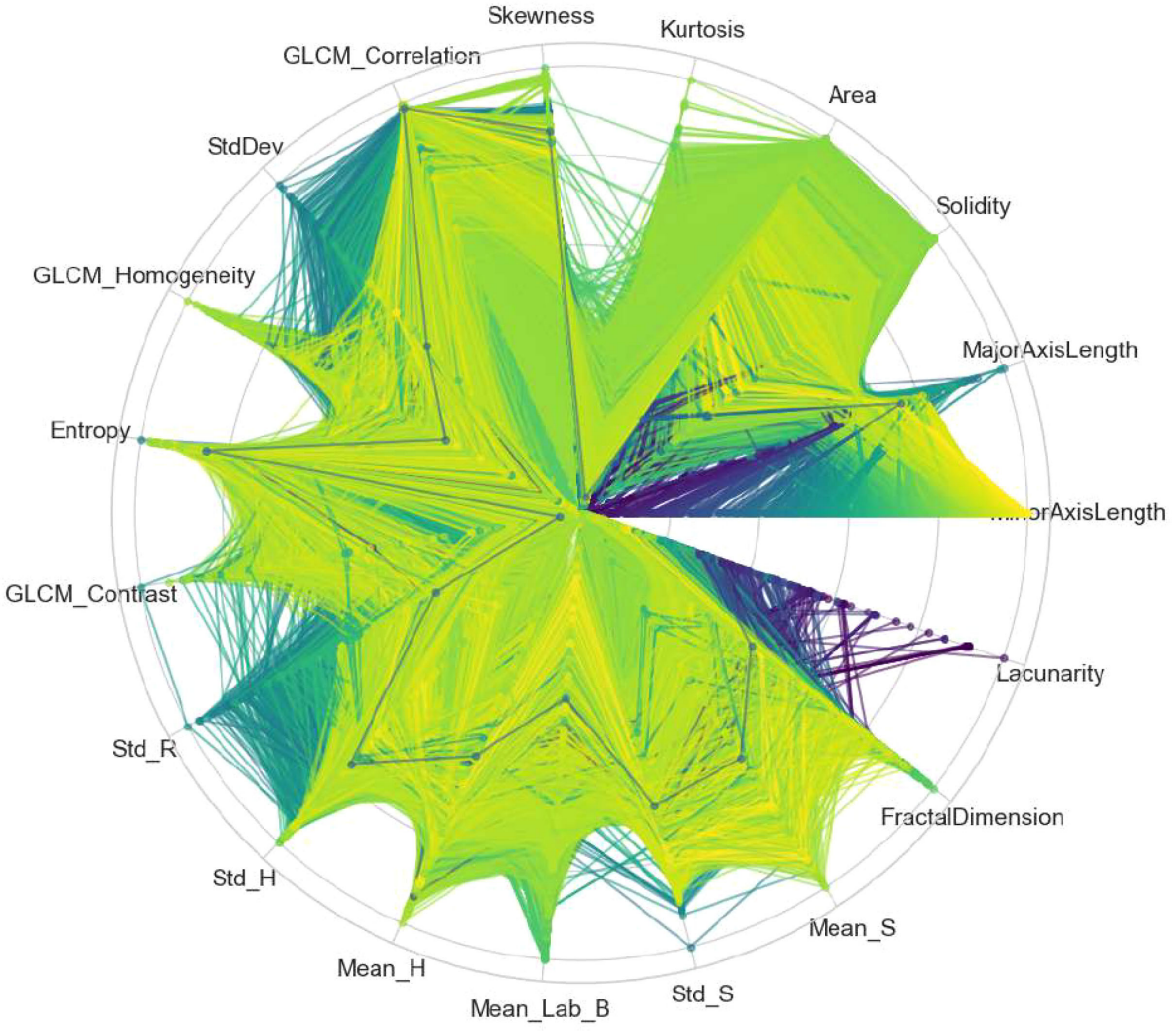
Radial coordinate plot of HCF, showing feature distributions and class-wise variation in a circular representation. HCF, handcrafted feature.

The distribution and variation of the extracted handcrafted features are visualized using a parallel coordinates plot, as shown in **Figure 3**. In this plot, each vertical axis represents an individual feature, while each line corresponds to a sample across different classes. The variation in line patterns reflects differences in feature values among leukocyte categories, enabling a visual assessment of feature separability and discriminative capability. Further analysis of feature distributions is presented in **Figure 4** using a radial coordinate plot. In this visualization, each axis represents a feature, and the radial structure illustrates the relative magnitude of feature values across different classes. The resulting patterns provide insight into inter-class variation and highlight the discriminative characteristics of the handcrafted feature representation.

### 3.4 DenseNet201 features

Feature extraction is performed using the pre-trained DenseNet201 CNN (28). The DenseNet201 model was initialized with weights pre-trained on the ImageNet dataset, which contains over one million images across 1,000 classes. This pretraining enables effective transfer learning by providing rich hierarchical feature representations, which were leveraged in this study for extracting discriminative features from AML cell images. The dataset $D^{{\text{raw}}^{\prime }}$ consists of images *I_i_* and labels *l_i_*, where *i* ranges from 1 to *N*. Each image *I_i_* is resized to 224 × 224 pixels to ensure uniformity with the input layer of DenseNet201 and normalized using Norm_DenseNet_(*I_i_*) before feature extraction. DenseNet201, excluding its top classification layer, maps the pre-processed image to a deep feature space. The feature extraction function *f*_DN_ takes the input image *I_i_*, which has dimensions 1 × 224 × 224 × 3, and outputs a feature vector of dimensionality *d*. This process generates deep feature vectors that capture visual and color details essential for distinguishing between AML types (Equation 18).

(18) $$v_{i}=f_{\text{DN}}\left(I_{i}\right)\in {\mathbb{R}}^{d}$$

The global average subsampling operation summarized the visual activations from the final convolutional feature maps

(19) $$F_{j}=\frac{1}{H\times W}\sum_{h=1}^{H}\sum_{w=1}^{W}z_{j}\left(h,w\right)$$

where *z_j_*(*h*, *w*) denotes the activation at position (*h*, *w*) in the *j*th channel.

The extracted feature vectors from all *N* images were then compiled into a single feature matrix.

(20) $$V=\left[v_{1}^{T},v_{2}^{T},v_{3}^{T},\ldots ,v_{N}^{T}\right]\in {\mathbb{R}}^{N\times d}$$

where each row *v_i_* corresponds to the feature vector of the *i*th image. The class labels were stored in the vector *l_i_*.

(21) $$l_{i}=\left[l_{1},l_{2},l_{3},\ldots ,l_{i}\right]\in {\left\{1,\ldots ,C\right\}}^{N}$$

(22) $$V_{\text{DN}}=\left[v|l\right]\in {\mathbb{R}}^{N\times \left(d+1\right)}$$

### 3.5 MobileNetV2 features

MobileNetV2 is a lightweight CNN characterized by its high performance and low complexity that is used to extract features (29), meaning that its top classification layer is removed. The MobileNetV2 model was initialized with weights pre-trained on the ImageNet dataset, enabling efficient transfer learning by capturing both low-level and high-level visual features while maintaining computational efficiency. In this work, the convolutional base of the model is used as a feature extractor, and the extracted features are subsequently utilized within the proposed TCMA-Net classification framework.

This architecture has been selected because it has been demonstrated to isolate the salient features that are needed to correctly classify AML. It works with a balanced set represented by $D^{{\text{raw}}^{\prime }}$, which consists of images *I_i_* and labels *l_i_* ranging from 1 to *N*. All the images *I_i_* were uniformly resized to 224 × 224 pixels to ensure consistency with the input layer of the MobileNetV2 model.

The resized image *I_i_* undergoes normalization Norm_MobileNet_(*I_i_*) to adjust it for model input. The feature extraction process is represented by *f*_MN_(*I_i_*), where *I_i_* with dimensions 1 × 224 × 224 × 3 is mapped to a feature vector of dimensionality *d*. This process outputs a deep feature vector for each image via MobileNetV2 (Equation 23).

(23) $$v_{i}=f_{\text{MN}}\left(I_{i}\right)\in {\mathbb{R}}^{d}$$

The global average pooling operation, herein, pooled the visual activations on the terminal convolutional feature maps together into one image-specific vector, as described in Equation 19. The resultant feature vectors of each image were then grouped into a feature matrix, as is described in Equation 20. Each row *v_i_* is a feature vector of the *i*th image, as explained in Equation 21. The class labels were stored in the vector *l_i_*. After the extraction of the feature phase, feature selection was conducted using the chi-square test (30), which is a statistical method used to determine the relevance of each predictor variable with respect to the target categories. The broad aim of this process was to keep the most important attributes to use in classification, hence cutting off the qualities that were inconsequential.

First, the class labels *l_i_* were encoded into numeric values using LabelEncoder if they were classified (Equation 24).

(24) $$l_{\text{encoded}}=\text{LabelEncoder}\left(l_{i}\right)$$

Next, the feature matrix *V* was scaled using MinMaxScaler to ensure that all features contribute equally to the chi-square test (Equation 25).

(25) $$V_{\text{scaled}}=\text{MinMaxScaler}\left(V\right)$$

The chi-square test was then applied to assess the relationship between each feature and the class labels, where *V*_scaled_ is the scaled feature matrix after applying MinMax scaling (Equation 26).

(26) $$x^{2}\left(V_{\text{scaled}},l_{\text{encoded}}\right)=\left(x_{\text{score}}^{2},\text{P}\_\text{values}\right)$$

Features with *p*-values below a threshold of 0.05 were regarded as statistically significant. The selected features are represented as the set of features *v* for which the corresponding *p*-value *P_i_* is less than 0.05. Finally, the selected features were extracted from the scaled feature matrix *V*_scaled_, and the corresponding label vector *l_i_* was added to form the final dataset. This process is represented by the selection of *V*_selected_ from the scaled feature matrix, which includes only the features that were selected. The final selected feature dataset is saved (Equation 27).

(27) $$D_{\text{selected}}=\left[V_{\text{selected}}|l_{i}\right]\in {\mathbb{R}}^{N\times \left(d_{\text{selected}}+1\right)}$$

where *d*_selected_ is the number of features selected after implementing the chi-square test.

### 3.6 HCF + MobileNetV2 features

The feature model results from the HCF extraction framework and the MobileNetV2 feature extraction framework were fused to form a hybrid feature representation. The target of this fusion was to improve AML cell classification accuracy.

The final feature vector from the HCF extraction process is (Equation 28)

(28) $$F_{\text{fused}}=[F_{\text{shape}}\;∥\;F_{\text{texture}}\;∥\;F_{\text{color}}\;∥\;F_{\text{fractal}}]$$

where *F*_shape_, *F*_texture_, *F*_color_, and *F*_fractal_ represent the shape, texture, color, and fractal features, respectively, and ∥ denotes the feature merging.

The final feature vector from the MobileNetV2 feature extraction process is (Equation 29)

(29) $$V_{\text{MN}}=\left[l_{1},l_{2},l_{3},\ldots ,l_{i}\right]\in {\left\{1,\ldots ,C\right\}}^{N}$$

where each *l_i_* denotes a deep feature value obtained from the global average pooling layer, *c* represents the total number of classes, and *N* denotes the number of samples. Both features were combined to obtain a hybrid representation (Equation 30).

(30) $$V_{\text{HCF}+\text{MN}}=F_{\text{fused}}\;∥\;V_{\text{MN}}$$

where $V_{\text{HCF}+\text{MN}}$ denotes the hybrid feature vector of dimensionality (*m* + *n*), with *m* and *n* corresponding to the number of handcrafted and MobileNetV2 features, respectively.

Before applying the technique of feature selection, both features were normalized using the MinMax scaling method to ensure that all features contributed equally (Equation 31).

(31) $${\tilde{V}}_{\text{HCF}+\text{MN}}^{\left(j\right)}=\frac{V_{\text{HCF}+\text{MN}}^{\left(j\right)}-\min\left(V_{\text{HCF}+\text{MN}}^{\left(j\right)}\right)}{\max\;\left(V_{\text{HCF}+\text{MN}}^{\left(j\right)}\right)-\min\;\left(V_{\text{HCF}+\text{MN}}^{\left(j\right)}\right)}$$

for each feature *j* in the hybrid feature set. Then, feature selection was performed using the chi-square test to assess the dependency between each feature and the class labels (Equation 32).

(32) $$x_{j}^{2}=\sum_{r=1}^{c}\frac{{\left(O_{jr}-E_{jr}\right)}^{2}}{E_{jr}}$$

where *O_jr_* and *E_jr_* represent the observed and expected frequencies, respectively, of feature *j* for class *r*, and *c* is the total number of classes.

Features with *p<* 0.05 were kept as significant features for the final set (Equation 33).

(33) $$V_{\text{selected}}=\left\{{\tilde{V}}_{\text{HCF}+\text{MN}}^{\left(j\right)}|P_{j}<0.05\right\}$$

The resulting *V*_selected_ represents important hybrid features combining handcrafted morphological details and deep visual patterns from MobileNetV2, used later for classification to improve both efficiency and understanding.

### 3.7 Proposed HADNet features

In this research, the final features from the output of HCF and DenseNet201 were fused to createa comprehensive and highly representative feature space efficient in improving the classificationaccuracy of AML leukemia cells, and then, a novel architecture named HADNet was proposed forresilient AML leukemia cell feature extraction (**Algorithm 2**).

The final feature representation from the HCF extraction process is denoted as *F*_fused_, as described in Subsection 3.3 in Equation 17. Similarly, the DenseNet201 feature extraction process is given by *V*_DN_, as defined in Subsection 3.4 in Equation 22.

To construct the proposed HADNet feature space, (Equation 34)

(34) $$V_{\text{HADNet}}=F_{\text{fused}}\;∥\;V_{\text{DN}}$$

where $V_{\text{HADNet}}\in {\mathbb{R}}^{N\times \left(m+d\right)}$, with *m* being the total number of handcrafted features.

Before feature selection, scaling is applied to each feature to ensure uniformity across HCF and deep features (Equation 35).

(35) $${\tilde{V}}_{\text{HADNet}}^{\left(j\right)}=\frac{V_{\text{HADNet}}^{\left(j\right)}-\min\;\left(V_{\text{HADNet}}^{\left(j\right)}\right)}{\max\;\left(V_{\text{HADNet}}^{\left(j\right)}\right)-\min\left(V_{\text{HADNet}}^{\left(j\right)}\right)}$$

Feature selection was then performed on the scaled hybrid feature matrix. ${\tilde{V}}_{\text{HADNet}}$ used the chi-square test as denoted in Equation 32.

Features with *P_j_*< 0.05 were recognized as important and kept in the final HADNet feature set (Equation 36).

(36) $$V_{\text{HADNet}\_\text{Sel}}=\left\{{\tilde{V}}_{\text{HADNet}}^{\left(j\right)}|P_{j}<0.05\right\}$$

In another approach, if a fixed number of features were needed, the top-k features were selected based on the chi-square statistics (Equation 37).

(37) $$V_{\text{HADNet}}^{k}=\arg\;{\text{top}}_{k}\;x_{j}^{2}$$

Hence, the resulting *V*_HADNet_ or $V_{\text{HADNet}}^{k}$ captures both morphological features and deep visual information, providing a strong base for the accurate classification of AML leukemia cells.

### 3.8 TCMA-Net classification

The proposed TCMA-Net architecture (**Algorithm 3**) combines a CNN and a ViT to jointly capture local spatial patterns and global contextual dependencies among extracted descriptors. In this study, the classifier did not directly operate on raw microscopic images. Instead, the input to TCMA-Net was derived from feature vectors obtained from different feature extraction strategies (e.g., handcrafted descriptors, CNN-based semantic features, or their fused representations). Each sample is represented by a numerical feature vector, which is first standardized and then transformed into a two-dimensional pseudo-image representation to enable compatibility with deep learning architectures.

Algorithm 2 **Proposed HADNet feature extraction process.**

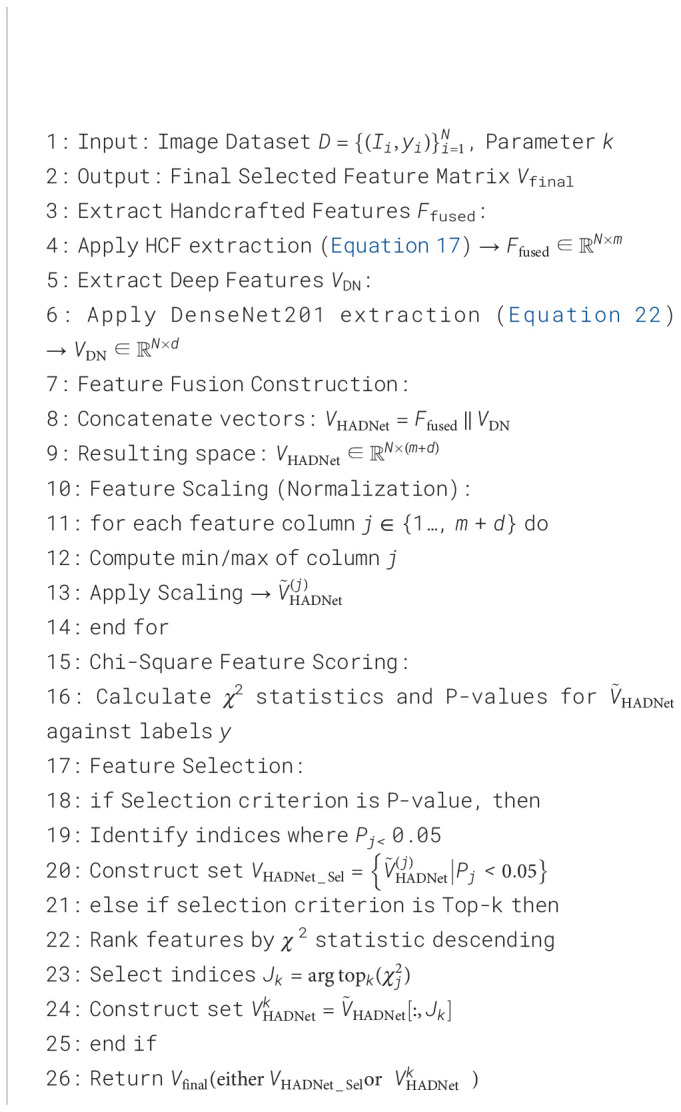

Specifically, let $x_{i}\in {\mathbb{R}}^{d}$ denote the feature vector of the *i*th sample containing *d* extracted features. To construct a spatial representation, the feature vector was reshaped into a square matrix of size *m* × *m*, where $m=\left[\sqrt{d}\right]$. If *m*^2^ > *d*, zero padding was applied to complete the matrix structure. The resulting matrix was treated as a pseudo-image and resized to a fixed spatial resolution of 32 × 32 using interpolation. This transformation preserves the relative relationships among features while allowing CNN and transformer layers to learn spatial interactions between descriptors. The resulting pseudo-image is therefore denoted as $I_{i}\in {\mathbb{R}}^{32\times 32\times 1}$ and used as input for both CNN and ViT branches.

#### 3.8.1 CNN branch

The CNN branch consists of three convolutional blocks designed to learn local spatial interactions between feature elements in the pseudo-image representation. Each block contains two convolutional layers followed by batch normalization, max-pooling, and dropout regularization.

The first convolutional layer extracts low-level spatial patterns from the pseudo-image representation. The convolution operation followed by a Rectified Linear Unit (ReLU) activation function is expressed as Equation 38

(38) $$U_{1}=\rho \left(K_{1a}*I_{i}+b_{1a}\right)$$

where *K*_1_*_a_* denotes the convolution kernel, *b*_1_*_a_* represents the bias term, *ρ*(·) is the ReLU activation function, and ∗ denotes convolution. A second convolutional layer further refines the extracted spatial representations, (Equation 39)

(39) $$U_{2}=\rho \left(K_{1b}*U_{1}+b_{1b}\right)$$

Batch normalization is then applied to stabilize feature distributions, followed by max-pooling to reduce spatial resolution and dropout to prevent overfitting. These operations produce intermediate feature representations that are progressively refined in subsequent convolutional blocks, where the number of filters increases from 32 to 64 and finally to 128.

After the final convolutional block, the resulting feature map is flattened into a vector representation (Equation 40).

(40) $$v_{\text{CNN}}=\text{vec}\left(C_{3}\right)\in {\mathbb{R}}^{D_{\text{cnn}}}$$

This vector is passed through a dense layer with 256 neurons and dropout regularization to produce the final CNN representation (Equation 41).

(41) $$h_{\text{CNN}}=\text{Drop}*0.2\left(\rho \;\left(W*\text{cnn}v_{\text{CNN}}+b_{\text{cnn}}\right)\right)$$

#### 3.8.2 ViT branch

The ViT branch captures global contextual relationships among the spatially arranged feature descriptors. The pseudo-image *I_i_* is divided into non-overlapping patches. For an input resolution of 32 × 32 and a patch grid of 4 × 4, each patch has spatial dimensions of 8 × 8. The patch extraction operation is defined as Equation 42

(42) $$P=\text{Π}\left(I_{i}\right)$$

where Π(·) denotes the patch partitioning operator.

Each patch is projected into a higher-dimensional embedding space using a learnable projection matrix, (Equation 43)

(43) $$Z_{0}=PE+b_{e}$$

where *E* denotes the embedding matrix and *b_e_* represents the bias vector. Positional embeddings are added to preserve the spatial order of patches. The embedded sequence is processed through transformer encoder layers composed of multi-head self-attention and feed-forward networks. The attention mechanism is expressed as Equation 44

(44) $$A_{t}=\text{MHA}\;\left(\text{LN}\left(Z_{t-1}\right),\text{LN}\left(Z_{t-1}\right),\text{LN}\left(Z_{t-1}\right)\right)$$

where LN denotes layer normalization and MHA represents the multi-head attention operation. These transformer layers model long-range dependencies between feature regions. After the transformer encoder layers, the sequence representation is flattened, (Equation 45)

(45) $$v_{\text{ViT}}=\text{vec}\left(Z_{T}\right)$$

and passed through a dense layer with dropout to obtain the final ViT representation (Equation 46)

(46) $$h_{\text{ViT}}=\text{Drop}*0.2\;\left(\rho \;\left(W*\text{ViT}v_{\text{ViT}}+b_{\text{ViT}}\right)\right)$$

Algorithm 3 **TCMA-Net classification model.**

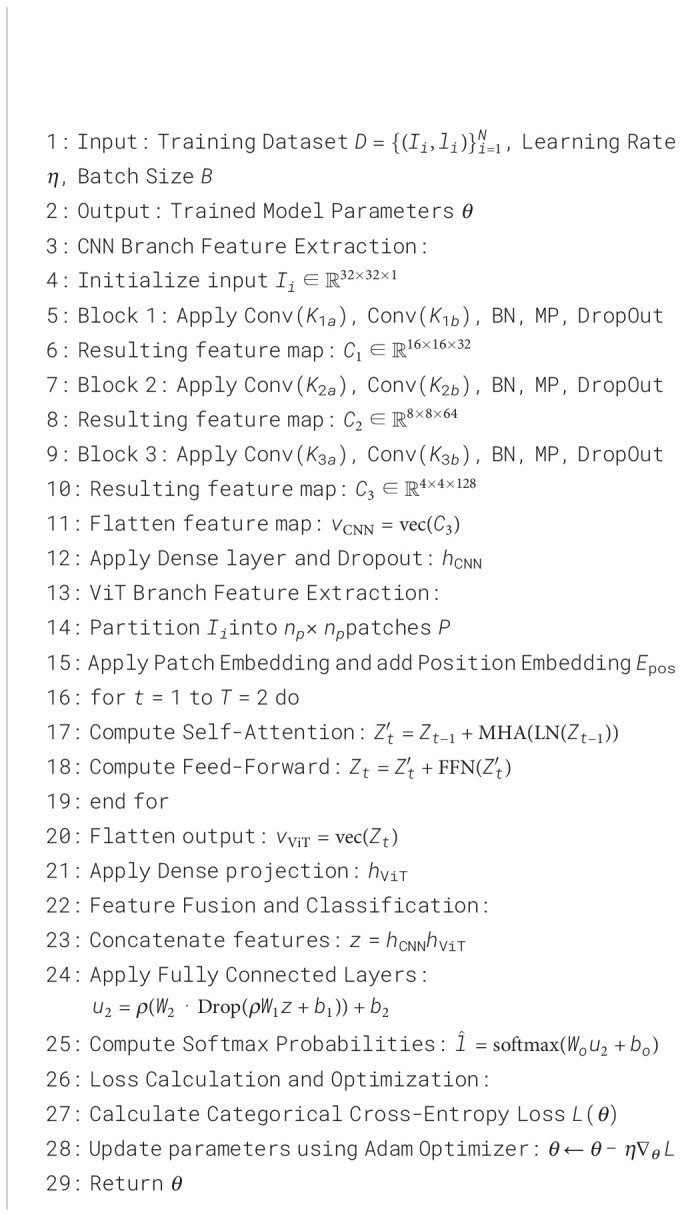

#### 3.8.3 Fusion mechanism

The feature representations obtained from the CNN and ViT branches are concatenated to form a unified representation, (Equation 47)

(47) $$z=h_{\text{CNN}},||,h_{\text{ViT}}$$

This fused vector integrates both local spatial patterns and global contextual relationships learned from the pseudo-image representation. The fused features are then passed through two fully connected layers, (Equations 48, 49)

(48) $$u_{1}={\text{Drop}}_{0.2}\left(\rho \left(W_{1}z+b_{1}\right)\right)$$

(49) $$u_{2}=\rho \left(W_{2}u_{1}+b_{2}\right)$$

The final classification layer produces logits, (Equation 50)

(50) $$s=W_{o}u_{2}+b_{o}$$

which are converted into class probabilities using the softmax function, (Equation 51)

(51) $$\hat{l}=\text{softmax}\left(s\right)$$

The network is trained using the categorical cross-entropy loss, (Equation 52)

(52) $$L\left(\theta \right)=-\frac{1}{N}\sum_{i=1}^{N}\sum_{c=1}^{C}l_{i,c}\text{log }({\hat{l}}_{i,c})$$

Model parameters *θ* are optimized using the Adam optimizer with learning rate *η* = 10^−4^, (Equation 53)

(53) $$\theta \leftarrow \theta -\eta \nabla_{\theta }L$$

### 3.9 Training implementation details

All experiments were implemented in Python using the TensorFlow and Keras frameworks. The extracted fused feature vectors were normalized using the StandardScaler and converted into pseudoimage representations to enable compatibility with the CNN–Transformer architecture. Specifically, each feature vector was reshaped into a square grid using zero padding when necessary and resized to a fixed resolution of 32 × 32 to form a single-channel grayscale input. The dataset was divided using an 80:20 stratified train–test split, and class labels were encoded using label encoding followed by one-hot encoding. The proposed TCMA-Net consists of a CNN and a ViT branch designed to capture both local spatial patterns and global contextual relationships. The model was trained using the Adam optimizer and categorical cross-entropy loss. The complete set of hyperparameter settings used for training the model is summarized in **Table 3**. A formal hyperparameter sensitivity analysis was not conducted due to computational constraints; however, the selected hyperparameters were determined based on empirical validation and commonly adopted configurations in related deep learning studies. The chosen settings, including learning rate, batch size, and optimizer, provided stable convergence and consistent performance across all experiments, as reflected in the reported results. Model performance was evaluated using multiple metrics, including accuracy, precision, recall, F1-score, Matthews correlation coefficient (MCC), and area under the curve (AUC). All experiments were conducted on a workstation equipped with an NVIDIA GeForce RTX 3080 GPU, 10GB VRAM, and a 13th-Generation Intel Core i9 processor.

**Table 3:** Hyperparameter configuration used for training the TCMA-Net model.

Hyperparameter	Value
Input size	32 × 32 × 1
Patch grid	4 × 4
Patch size	8 × 8
Projection dimension	32
Transformer layers	2
Attention heads	2
CNN filters	32, 64, 128
Batch size	64
Epochs	100
Optimizer	Adam
Learning rate	1 × 10^−4^
Loss function	Categorical cross-entropy
Dropout rate	0.2

TCMA-Net, Tri block Convolutional Multi head Attention Network; CNN, convolutional neural network.

### 3.10 Performance evaluation metrics

To evaluate the performance of the proposed AML classification framework, several standard classification metrics were employed, including testing accuracy, precision, recall, F1-score, MCC, and AUC of the receiver operating characteristic (ROC) curve. These metrics provide a comprehensive assessment of classification performance by measuring different aspects of prediction quality.

Accuracy measures the overall correctness of the classification model and is defined as Equation 54

(54) $$Accuracy=\frac{TP+TN}{TP+TN+FP+FN}$$

where *TP*, *TN*, *FP*, and *FN* denote true positives, true negatives, false positives, and false negatives, respectively. Precision measures the proportion of correctly predicted positive samples among all predicted positives and is given by Equation 55

(55) $$Precision=\frac{TP}{TP+FP}$$

Recall (also known as sensitivity) evaluates the ability of the model to correctly identify positive samples and is defined as Equation 56

(56) $$Recall=\frac{TP}{TP+FN}$$

The F1-score represents the harmonic mean of precision and recall and is calculated as Equation 57

(57) $$F1=2\times \frac{Precision\times Recall}{Precision+Recall}$$

The MCC provides a balanced evaluation of classification performance, even for imbalanced datasets, and is expressed as Equation 58

(58) $$MCC=\frac{TP\times TN-FP\times FN}{\sqrt{\left(TP+FP\right)\left(TP+FN\right)\left(TN+FP\right)\left(TN+FN\right)}}$$

Additionally, two supplementary indicators were used to analyze prediction reliability: Error Alignment Ratio (EAR) and Feature Interpretation Stability (FIS). The EAR measures the proportion of misclassified samples relative to the total number of predictions and is defined as Equation 59

(59) $$EAR=\frac{N_{error}}{N_{total}}$$

where *N_error_* denotes the number of misclassified samples and *N_total_* represents the total number of samples. FIS measures the consistency between predicted and true labels and is defined as Equation 60

(60) $$FIS=\frac{1}{N}\sum_{i=1}^{N}\left|y_{i}-{\hat{y}}_{i}\right|$$

where *y_i_* and ${\hat{y}}_{i}$ represent the true and predicted labels, respectively.

Additionally, the AUC-ROC was used to evaluate the model’s ability to distinguish between classes across different decision thresholds. A higher AUC value indicates better discriminative capability of the classifier.

## 4 Experimental results and discussion

This section comprises a systematic comparison of the five feature extraction strategies, namely, HCF, DenseNet201, MobileNetV2, HCF + MobileNetV2, and the proposed HADNet, all fused with the new TCMA-Net classifier. All experiments were conducted on the SA3 balanced dataset with a standard 80:20 training and testing split and evaluated on a wide range of metrics. The findings show that there is a gradual yet steady increase in classification stability and accuracy, which depicts the advantage of more refined and merged feature representations.

### 4.1 Handcrafted features-based experimental analysis

**Figure 5:**
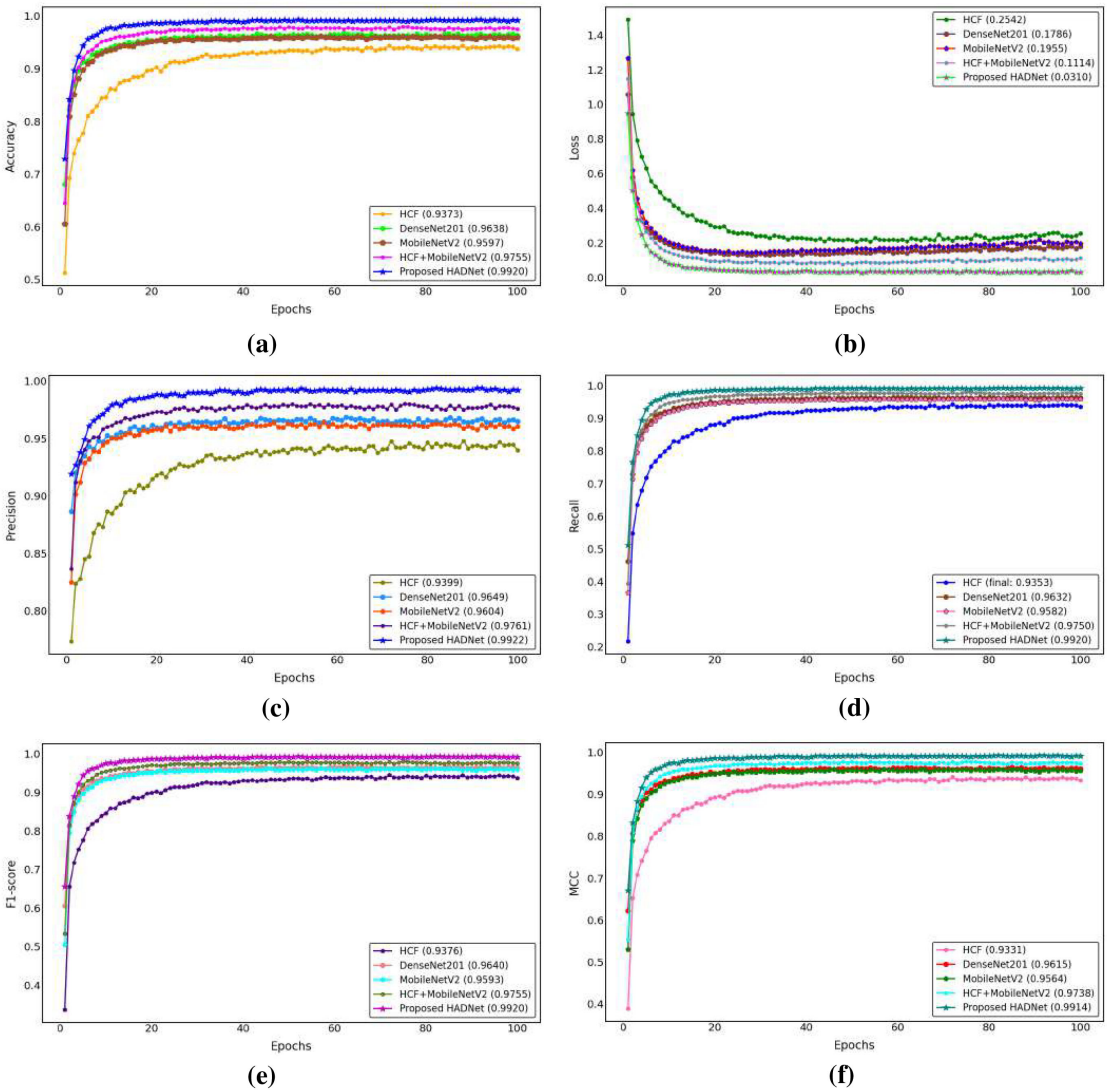
**(a)** Accuracy of experimental studies. **(b)** Loss graph. **(c)** Precision. **(d)** Recall of all studies. **(e)** Performance metric of F1-score. **(f)** The MCC curve graph. MCC, Matthews correlation coefficient.

**Figure 6:**
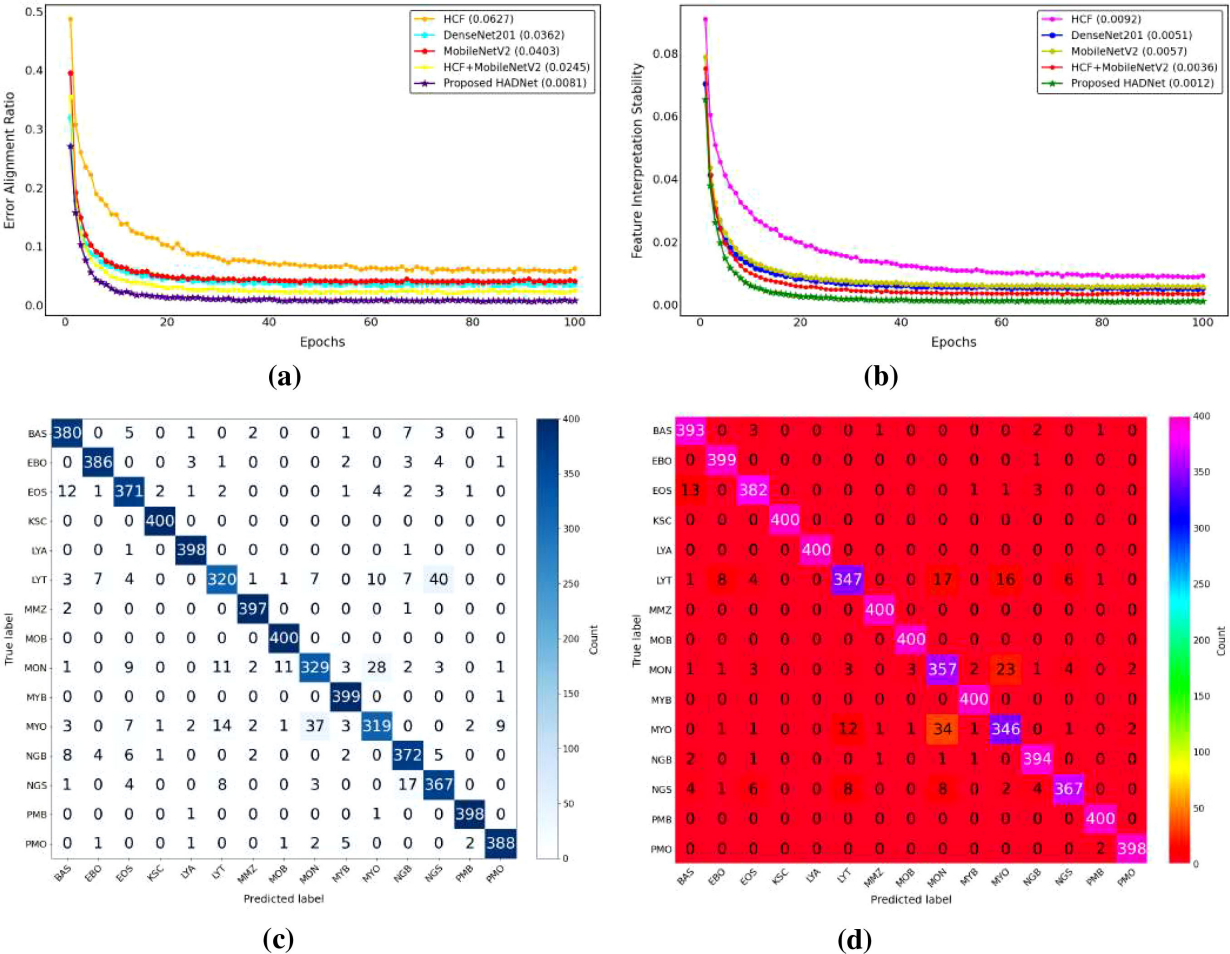
Evaluation and interpretability analysis of the proposed framework. **(a)** Experimental error ratio, **(b)** feature interpretation stability, **(c)** HCF-based confusion matrix, and **(d)** DenseNet201-based confusion matrix. HCF, handcrafted feature.

**Figure 7:**
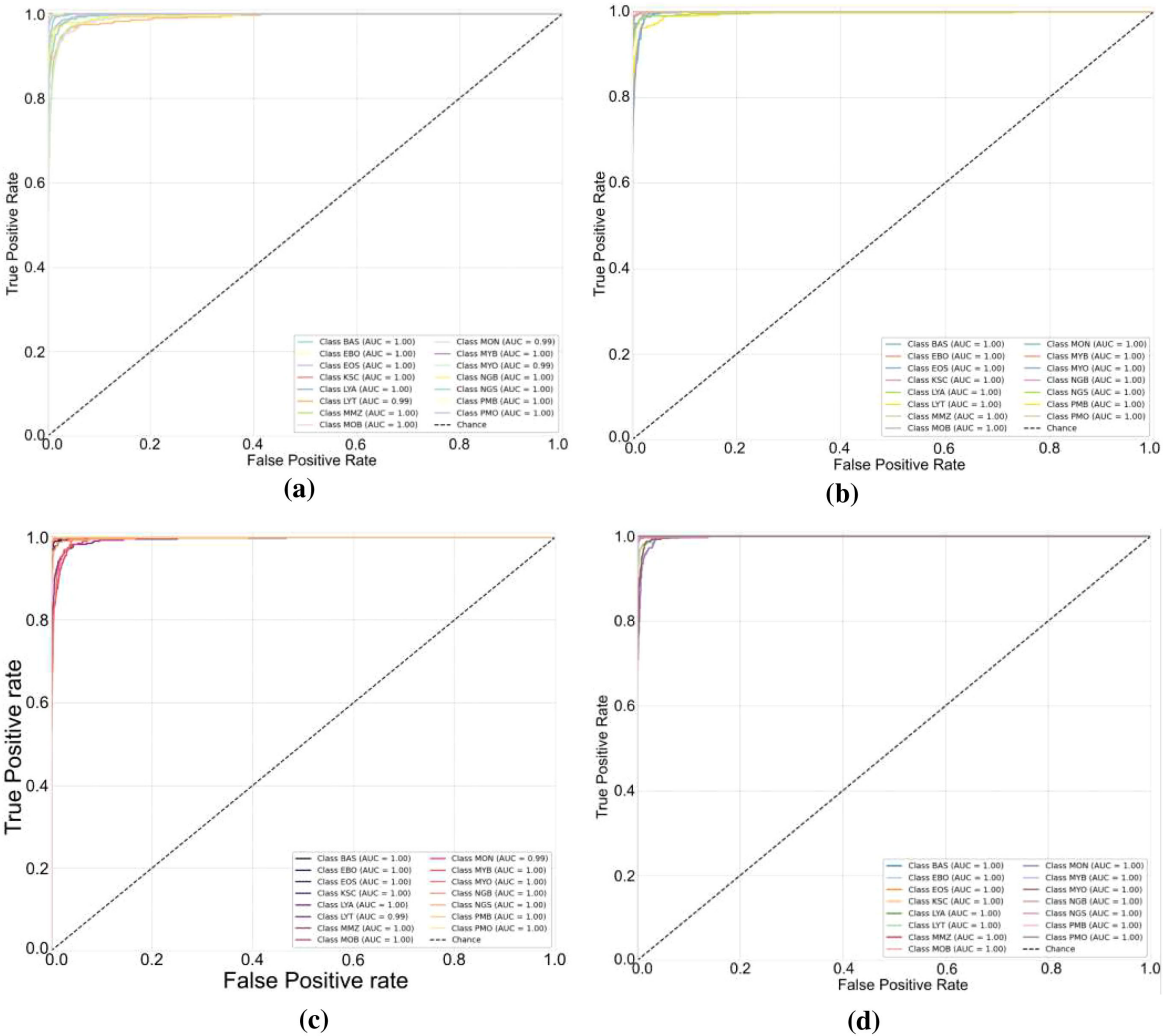
Receiver operating characteristic (ROC) curves for different feature representations: **(a)** HCF, **(b)** DenseNet201, **(c)** MobileNetV2, and **(d)** HCF + MobileNetV2. HCF, handcrafted feature.

**Figure 8:**
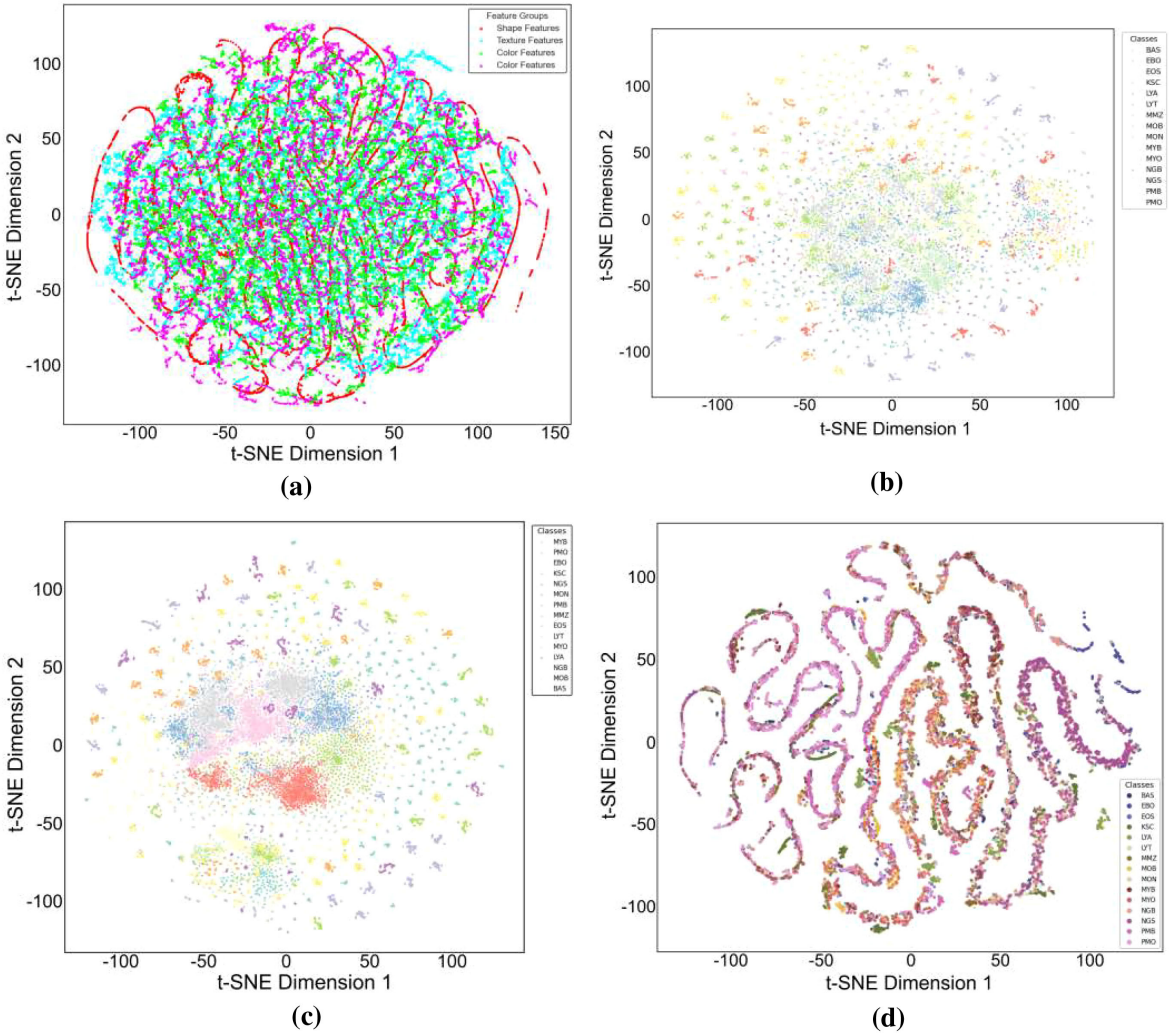
t-SNE visualizations of learned feature spaces: **(a)** HCF, **(b)** DenseNet201, **(c)** MobileNetV2, and **(d)** HCF + MobileNetV2. HCF, handcrafted feature.

**Figure 9:**
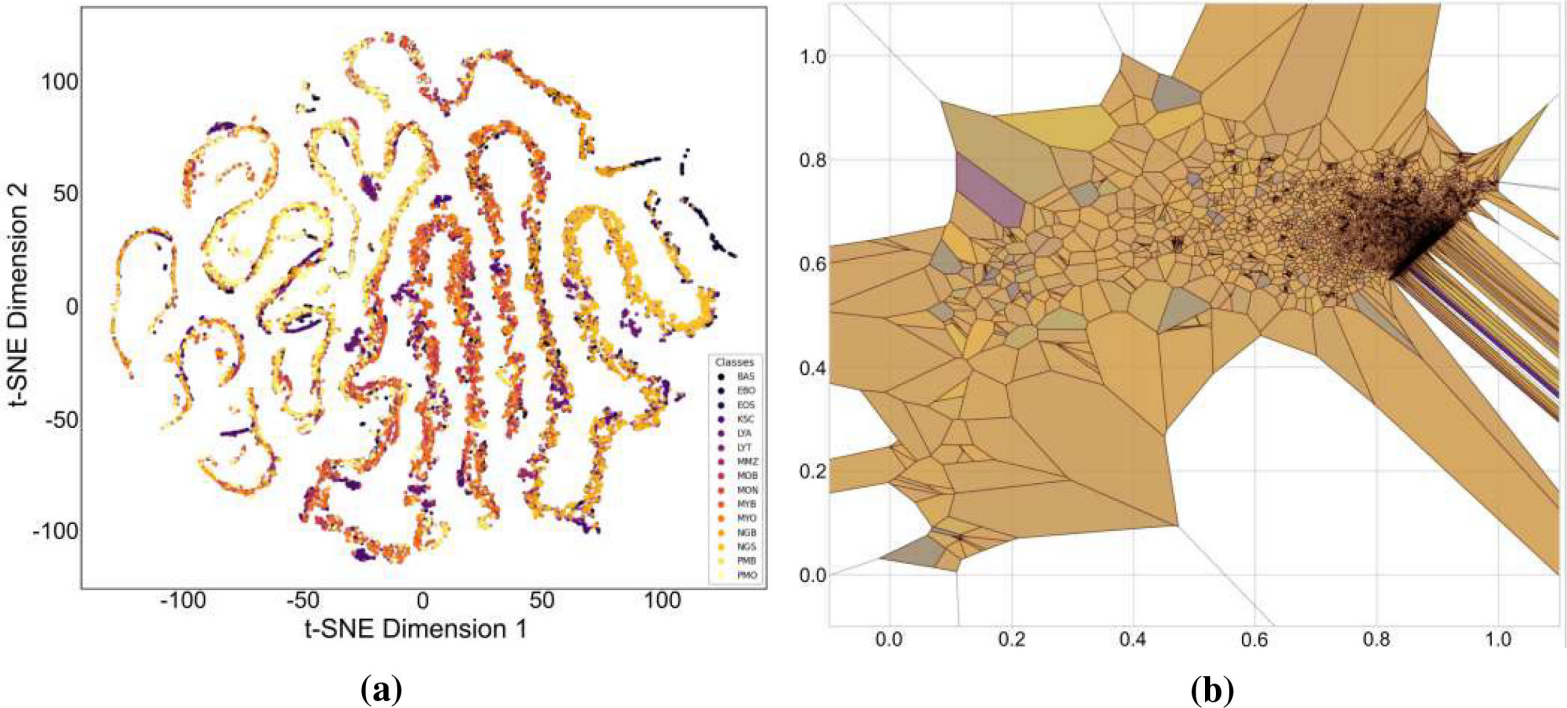
Feature space analysis of the proposed framework: **(a)** t-SNE visualization of HADNet features and **(b)** Voronoi diagram of handcrafted features.

The baseline experiment used HCF and TCMA-Net classifier to determine the initial benchmark of AML cell recognition. **Figure 5a** reveals a testing accuracy of 0.9373, and **Figure 5b** displays a testing loss value of 0.2542, indicating a consistent convergence and encoding of vital morphological, color, and texture features. The performance metrics, such as testing precision in **Figure 5c**, testing recall in **Figure 5d**, and testing F1-score in **Figure 5e**, showed balanced predictive behavior of leukocyte types. Moderate consistency and robustness were reflected through reliability values, including MCC in testing **Figure 5f**, testing the EAR in **Figure 6a**, and FIS in **Figure 6b**. **Figure 6c** represents a confusion matrix of the testing set that reveals some misclassifications between similar leukocyte subtypes and reveals that it is not easy to distinguish subtle variations in cytology with HCF only, whereas the testing ROC curve in **Figure 7a** indicates that HCF allows strong discriminative performance to baseline leukocyte classification, irrespective of how limited the depth of the features is. There was overlap between clusters in the t-distributed Stochastic Neighbor Embedding (t-SNE) visualization in **Figure 8a** and Voronoi tessellation in **Figure 9b**, which signifies a lack of separability of similar categories. This shows that handcrafted characteristics can only hold superficial visual patterns, and no semantic depth is observed to portray intricate cytological variations. On the whole, the experiment was a good starting point, and it confirmed the validity of classical descriptors of simple discrimination and the necessity of more profound, context-sensitive approaches to feature extraction that were discussed in further experiments.

### 4.2 Deep CNN-based feature experimental evaluation

**Figure 10:**
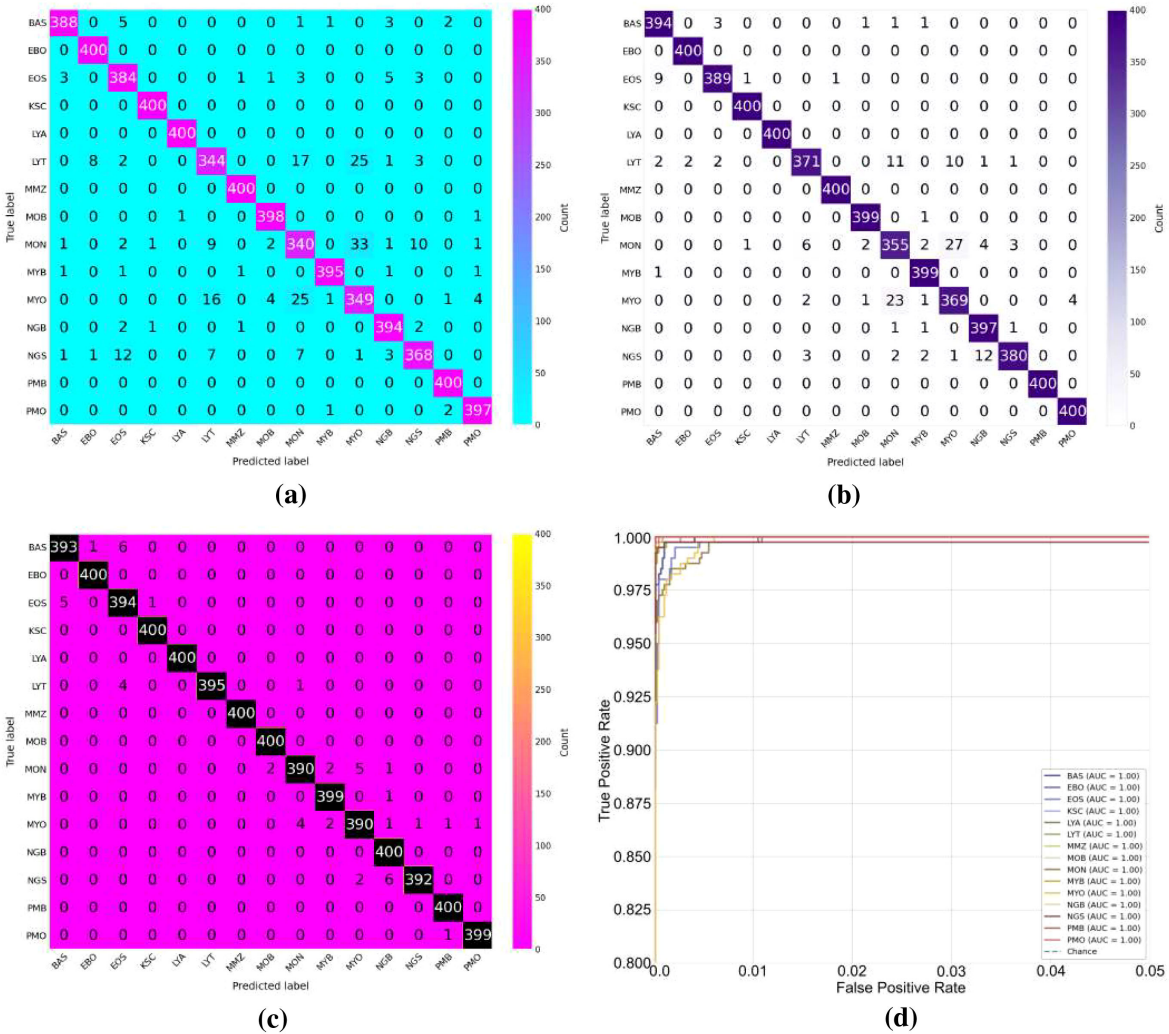
Comparative evaluation of classification performance. **(a)** MobileNetV2 confusion matrix, **(b)** HCF + MobileNetV2 confusion matrix, **(c)** HADNet confusion matrix, and **(d)** ROC curve of the proposed HADNet model. HCF, handcrafted feature; ROC, receiver operating characteristic.

To overcome the drawbacks of the use of handcrafted (HC) descriptors and, accordingly, to improve the discriminative representation of AML cell imagery, DenseNet201 and MobileNetV2 were used as two pre-trained deep convolutional frameworks to extract features. Further classification followed the TCMA-Net framework. The DenseNet201-based model, which predicts with an accuracy of testing 0.9638 as shown in **Figure 5a**, and a loss of 0.1786 as seen in **Figure 5b**, similarly, proved that it is not only converging steadily but also significantly out-of-sample learning features compared to HC alternatives. Similarly, the model based on MobileNetV2 achieved a testing accuracy of 0.9597 and a testing loss of 0.1955, indicating the strong performance along with a minimized computational load. These results highlight the value of the densely linked topology of DenseNet201 that encourages the reuse of features and the gradual feature preservation, and the inverted residual scheme of MobileNetV2 that maintains a high degree of efficiency without any significant loss in accuracy. **Figure 5c** shows the precision curves, which highlight the correct recognition of testing classes. This is added by the testing recall patterns presented in **Figure 5d**, which have strong sensitivity. In the meantime, the F1-score plot of **Figure 5e** testifies to the balanced, consistent performance of the models of all categories of leukocytes. Reliability measures, including the testing MCC in **Figure 5f**, the EAR in **Figure 6a**, and the FIS in **Figure 6b**, collectively showed a higher testing consistency and a more consistent interpretation of features than when the results are done by hand. The confusion matrices in **Figure 6d** of DenseNet201 and **Figure 10a** of MobileNetV2 clearly show clarity in the mechanisms of classes and a significant decrease in the errors of misclassification in comparison to the HC baseline in the testing phase. Both matrices exhibit distinctively high diagonal dominance, and this indicates that the CNNs have indeed internalized discriminative features. DenseNet201, specifically, is characterized by a marginally better clustering with better overall accuracy. Simultaneously, the testing ROC curves shown in **Figures 7b** and **c** show that DenseNet201 has a large AUC than MobileNetV2, which supports strong discriminative power. Accordingly, both architectures are nearer to the upper left of the ROC space than the baseline, and the trajectory of DenseNet201 is getting closer to the ideal point, which corresponds to its high testing precision and low loss. **Figure 8b**, which shows the t-SNE visualization of DenseNet201, exhibits clusters in compact forms with small overlap, which implies a high level of inter-class separability. **Figure 8c**, which depicts MobileNetV2 features, in turn, represents sharp but blurrier groupings, a reflection of the defining trade-off that representational profundity is in focus and computational expediency as the solution. Both these CNN-based experiments support the effectiveness of deep hierarchical feature representation to distinguish AML cells. DenseNet201 and MobileNetV2 were selected as feature extraction backbones due to their complementary architectural properties and effectiveness demonstrated in the ablation study. DenseNet201 utilizes dense connectivity, which promotes feature reuse and strengthens gradient flow, making it highly effective for capturing complex morphological patterns in medical images. In contrast, MobileNetV2 employs depthwise separable convolutions and inverted residual blocks, offering a lightweight architecture with reduced computational cost while maintaining strong representational capability. As shown in **Table 4**, DenseNet201 and MobileNetV2 individually achieve strong performance, and their integration further improves classification accuracy. Therefore, these models were chosen as a balanced combination of performance and efficiency for the proposed hybrid feature fusion framework.

**Table 4:** Ablation study evaluating different feature extraction strategies combined with the TCMA-Net classifier.

Feature strategy	Accuracy (%)	Precision (%)	Recall (%)	F1-score (%)
HCF + TCMA-Net	93.73	93.45	93.60	93.52
DenseNet201 + TCMA-Net	96.38	96.10	96.25	96.17
MobileNetV2 + TCMA-Net	95.97	95.70	95.85	95.77
(HCF + MobileNetV2) + TCMA-Net	97.55	97.30	97.40	97.35
HADNet + TCMA-Net (Proposed)	99.20	99.22	99.18	99.20

TCMA-Net, Tri block Convolutional Multi head Attention Network; HCF, handcrafted feature.

### 4.3 Hybrid feature-based evaluation of TCMA-Net

To improve the discriminative AML classification performance, hybrid feature representations were created, which merged both handcrafted and deep features. Two fusion-based models were considered—the HCF + MobileNetV2 hybrid and the proposed HADNet—both of which were categorized with the TCMA-Net model. The HCF + MobileNetV2 ensemble had a testing accuracy of 0.9755 as seen in **Figure 5a** and a testing loss of 0.1114 revealed in **Figure 5b**, which is an encouraging enhancement compared to single feature models. The model was successfully used to combine handcrafted morphological cues with the profound spatial attributes of MobileNetV2 to extract low-level cellular geometries and the high-level contextual semantics. The precision trends in **Figure 5c** support a high level of classification accuracy going along with the recall trends in **Figure 5d** and the patterns of the F1-score in **Figure 5e**, hence justifying a balanced predictive performance on leukocyte testing categories. Reliability indicators, with MCC shown in **Figure 5f**, the EAR discussed in **Figure 6a**, and the FIS shown in **Figure 6b**, exhibit better consistency and interpretability in the testing set. The confusion matrix shown in **Figure 10b** of the HCF + MobileNetV2 model shows very few misclassifications, showing that HCF, combined with deep space cues, effectively addressed the uncertainties in the constitutive models. **Figure 7d** shows the ROC curve of HCF + MobileNetV2, which has a large testing AUC, and the curves are concentrated close to the upper left corner, which further supports the idea of higher discriminative ability of the model. The t-SNE plot in **Figure 8d** demonstrates tight, well-separated clusters and hence justifies increased inter-class distinctiveness compared to the space of features generated by MobileNetV2 alone.

Based on this, the current HADNet feature extraction architecture, which is DenseNet201 with HCF, reached a testing accuracy of 0.9920 as demonstrated in **Figure 5a** and a testing loss of 0.0310 as demonstrated in **Figure 5b**, representing better convergence and lower error. As shown in **Figure 5c**, the precision reveals correct discrimination of the classes, **Figure 5d** shows high recall performance in the testing set, and the patterns of the testing F1-score in **Figure 5e** demonstrate excellent behavior. The reliability indices, that is, the testing MCC in **Figure 5f**, the EAR in **Figure 6a**, and the FIS in **Figure 6b**, all testify to the remarkable stability of the given model. The confusion matrix of the HADNet methodology, as shown in **Figure 10c**, has almost complete diagonal domination with almost negligible off-diagonal values in the test set. This highlights the enhanced discriminative power of the combination of HCF and hierarchies of DenseNet201 feature representations. The testing ROC curve of HADNet, provided in **Figure 10d**, shows that the AUC is close to unity. This finding supports near-complete separation of classes and is the perfect example of the discriminative performance of the model. A t-SNE projection in **Figure 9a** shows almost perfect separation of classes, hence illustrating an extreme distinction between normal and leukemic cell populations. The hybrid experiments support the fact that the combination of HC descriptors and deep CNN (DCNN) representations exploits both morphological accuracy and semantic abstraction. Overall, HADNet is the most accurate, with the lowest error rates, and best generalizes across the subtypes of AML, the most reliable and resistant in the overall classification framework TCMA-Net.

### 4.4 Comparative analysis with ML and DL models

To further validate the effectiveness of the proposed HADNet–TCMA-Net framework, a comprehensive comparison was conducted with conventional ML models and state-of-the-art DL architectures. Traditional classifiers, including SVM and RF, were implemented using the same extracted HADNet feature set, while the DL baseline of ResNet50 was evaluated under identical training conditions. The comparison results are summarized in **Table 5**, demonstrating that the proposed method consistently outperforms both conventional and DL approaches on the primary dataset in terms of accuracy, precision, recall, and F1-score. The comparative analysis across multiple feature extraction strategies, baseline models, and evaluation metrics provides a comprehensive quantitative assessment of the proposed framework. The results consistently demonstrate that the integration of handcrafted and deep features through the HADNet representation, combined with the TCMA-Net classifier, leads to improved performance, robustness, and generalization across different experimental settings.

**Table 5:** TCMA-Net model comparison with baseline models.

Model	Accuracy	Precision	Recall	F1-score
SVM	0.9805	0.9804	0.9804	0.9804
Random Forest	0.9810	0.9809	0.9810	0.9809
ResNet50	0.9633	0.9645	0.9632	0.9636
Proposed TCMA-Net (HADNet)	0.9920	0.9922	0.9920	0.9920

TCMA-Net, Tri block Convolutional Multi head Attention Network; SVM, Support Vector Machine.

### 4.5 Ablation study

An ablation study was conducted to evaluate the contribution of different feature extraction strategies used in the proposed framework. Five configurations were investigated, including handcrafted features (HCF), DenseNet201 features, MobileNetV2 features, hybrid HCF + MobileNetV2 features, and the proposed HADNet feature fusion, all combined with the TCMA-Net classifier under identical training conditions. The comparative results are presented in **Table 4**. As shown, the HCF-based model achieved a testing accuracy of 93.73%, while deep feature representations using DenseNet201 and MobileNetV2 improved the performance to 96.38% and 95.97%, respectively. The hybrid HCF + MobileNetV2 configuration further increased the accuracy to 97.55%, demonstrating the advantage of integrating handcrafted and deep features. The proposed HADNet feature fusion achieved the best performance with a testing accuracy of 99.20%, confirming that combining handcrafted morphological descriptors with deep semantic representations significantly enhances AML classification performance.

### 4.6 External validation on the ALL dataset

**Figure 11:**
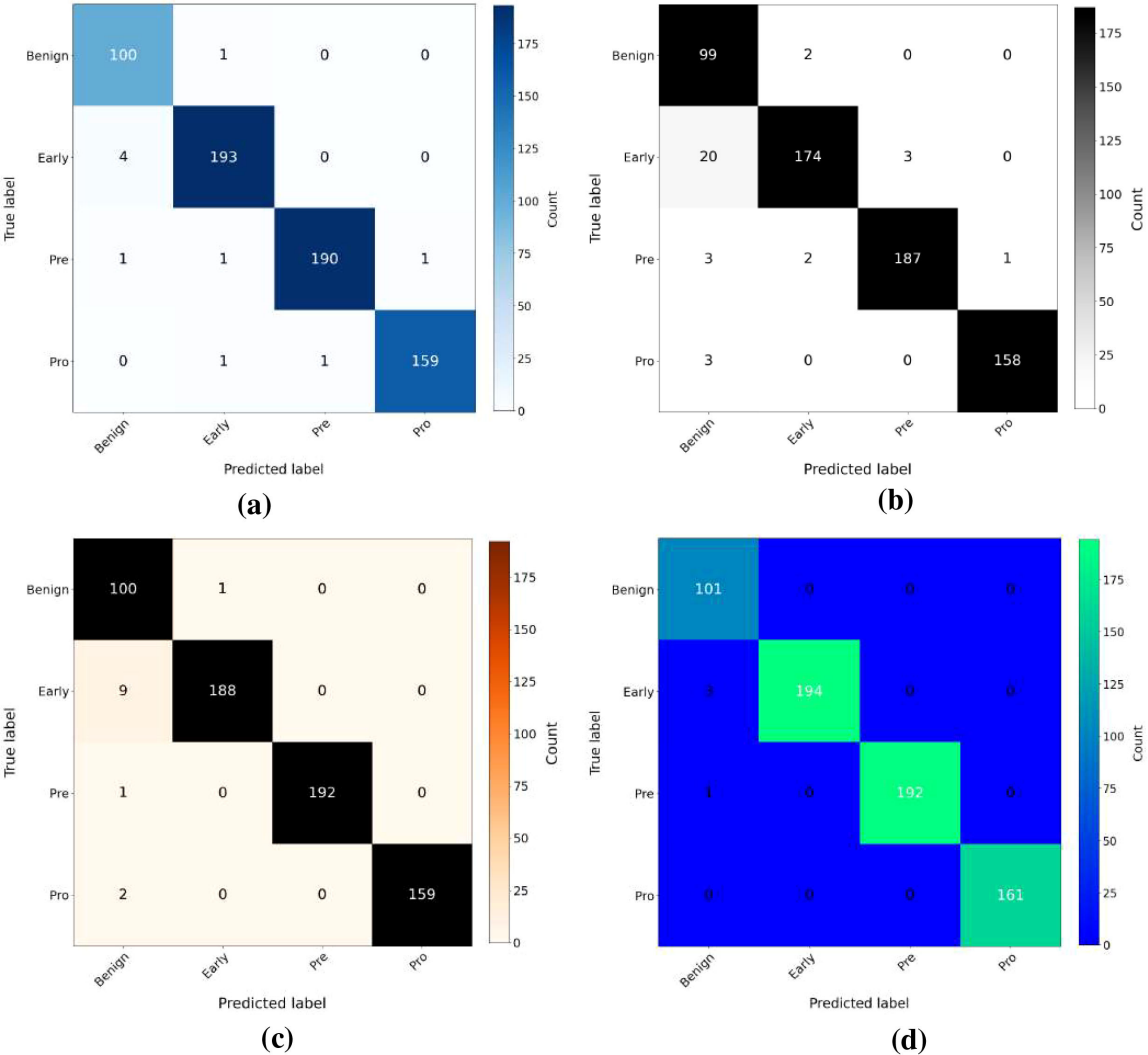
Comparative evaluation of classification performance. **(a)** ResNet50 confusion matrix, **(b)** RF confusion matrix, **(c)** SVM confusion matrix, and **(d)** TCMA-Net confusion Matrix. RF, Random Forest; SVM, Support Vector Machine; TCMA-Net, Tri block Convolutional Multi head Attention Network.

To demonstrate the robustness and generalization capability of the proposed framework, external validation was conducted using an independent dataset (ALL) obtained from a different source than the primary dataset, ensuring evaluation on unseen data distributions. This external validation ensures that the proposed HADNet feature fusion and TCMA-Net classifier are not biased toward a single dataset and can generalize effectively across different data domains. To maintain consistency with the primary experimental protocol, the dataset was first divided into training and testing subsets using an 80:20 stratified split, after which the proposed S3A method was applied exclusively to the training set to address class imbalance, while the testing set was kept completely unseen and unchanged to ensure unbiased evaluation. Following preprocessing, the same feature extraction pipeline employed in the primary dataset was utilized, where HCF and deep features extracted from DenseNet201 were fused to construct the HADNet feature representation for both training and testing sets. These fused features were then used as input for all classification models to ensure a fair and consistent comparison. Subsequently, models including SVM, RF, ResNet50, and the proposed TCMA-Net were trained and evaluated on the ALL dataset using the extracted HADNet features. The confusion matrix of ResNet50 is illustrated in **Figure 11a**, showing its classification performance across different classes. Similarly, the confusion matrix of the RF model is presented in **Figure 11b**, highlighting its prediction distribution. The confusion matrix of the SVM model is shown in **Figure 11c**, indicating its classification behavior and misclassification patterns. In contrast, the confusion matrix of the proposed TCMA-Net is depicted in **Figure 4**, demonstrating improved classification performance with stronger diagonal dominance. The quantitative results presented in **Table 6** demonstrate that the TCMA-Net model outperforms all baseline models across multiple evaluation metrics, achieving the highest classification accuracy and overall performance. This superiority is also evident from **Figure 11d**, where TCMA-Net shows fewer misclassifications compared to the baseline models. This consistent improvement across an independent dataset confirms that the proposed framework maintains high performance under varying data conditions, thereby demonstrating its robustness and strong generalization capability in cross-dataset evaluation scenarios. .

**Table 6:** Performance comparison of different models on the ALL dataset using HADNet features.

Model	Accuracy	Precision	Recall	F1-score
SVM	0.9801	0.98	0.98	0.98
Random Forest	0.9479	0.95	0.95	0.95
ResNet50	0.9847	0.9847	0.9847	0.9851
Proposed TCMA-Net	0.9939	0.9939	0.9939	0.9940

ALL, acute lymphoblastic leukemia; TCMA-Net, Tri block Convolutional Multi head Attention Network.

## 5 Discussion

The experimental findings prove the significant, gradual increase in the classification of AML cells with the increase in the sophistication of feature representations. The use of HCF created a strong base, which essentially summarized the key morphological and textural characteristics, but they had a limited ability to differentiate between different subtypes closely related in appearance. Deep CNN architectures and other methods significantly improved the level of feature discriminability through the need to learn complex spatial and contextual dependencies, and DenseNet201 achieved better accuracy due to its dense connectivity paradigm and the possibility of feature reuse. The hybrid fusion models, i.e., HCF + MobileNetV2 and the proposed HADNet, took the performance a notch higher by incorporating HCF and deep features, which succeeded in synthesizing fine-grained morphological information with the high-level semantically abstracted representations. HADNet was the most suitable one, as it showed the highest testing accuracy of 0.9920, the least testing loss, and the highest level of dividing the classes. This combination of qualities highlights its better generalization and strength. All these results support the assumption that the gradual enhancement and integration of feature representations, which leverage the advantageous aspects of distinct data modalities, when effectively integrated with the TCMA-Net classifier, ultimately result in a significant increase in accuracy, stability, and interpretability of AML cell classification. Although advanced visualization techniques such as Gradient-weighted Class Activation Mapping (Grad-CAM) can provide additional interpretability by highlighting discriminative regions in input images, the proposed framework already incorporates multiple qualitative evaluation methods, including t-SNE visualization, confusion matrices, and ROC curve analysis. These methods effectively demonstrate strong class separability, feature discriminability, and reliable model behavior, providing sufficient qualitative insight into the model’s decision-making process.

### 5.1 Comparison with other relevant studies

In comparison to recent studies in AML cell classification as given in **Table 7**, our proposed model outperforms existing methods. Yadav et al. (15) achieved an accuracy of testing 98.16% and a precision of 87.93% with 3SNet, while Elhassan et al. (21) reported 97% of testing accuracy and 98% of precision using their DCAE-CNN model. Erten et al. (31) achieved a testing set of 98.73% accuracy and 98.85% precision with ConcatNeXt, and Zhinin-Vera et al. (32) reported an accuracy above 90% and a precision of 92% with their DL model. Furthermore, Waqar et al. (33) proposed a ResNet50-based Hybrid Multi-Scale Contextual Attention Module (HMSCAM) framework for leukemia classification, achieving a multiclass accuracy of 93.5% on Peripheral Blood Smear (PBS) images. While these models performed well, the feature fusion approach in our model, combining both handcrafted and DL features, significantly enhances performance, leading to an accuracy of 99.20% and a precision of 99.22%. This fusion, coupled with the TCMA-Net classifier, allows our model to achieve superior generalization, effectively handling complex AML cell subtypes and providing more accurate and reliable classifications than these previous studies.

**Table 7:** Comparison with recent studies.

Authors	Study year	Proposed approach	Dataset	Precision	Accuracy
Yadav et al. (15)	2023	3SNet	AML	0.8793	0.9816
Elhassan et al. (21)	2023	DCAE-CNN	AML	0.9800	0.9700
Erten et al. (31)	2024	ConcatNeX t	Peripheral blood smear images	0.9885	0.9873
Zhinin-Vera et al. (32)	2024	DL Model	BMAS Images	0.92	*>*0.90
Waqar et al. (33)	2026	ResNet50 + HMSCAM	PBS images	–	0.935
Our study	2026	HADNet with TCMA-Net	AML	0.9922
Our study	2026	HADNet with TCMA-Net	PBS images (ALL)	0.9939	0.9939

TCMA-Net, Tri block Convolutional Multi head Attention Network; CNN, convolutional neural network; AML, acute myeloid leukemia.

### 5.2 Limitations and future work

Despite the strong performance of the proposed HADNet with the TCMA-Net framework, certain limitations remain. The evaluation is conducted on a limited number of datasets, which may not fully capture real-world clinical variability. Moreover, the hybrid feature fusion strategy, while improving discriminative capability, increases computational complexity compared to lightweight models, potentially affecting scalability. Additionally, advanced interpretability methods such as Grad-CAM were not incorporated, limiting localized insight into the model’s decision-making process. Future work will focus on validating the framework on larger and multi-center datasets to enhance generalization. The integration of explainability techniques, including Grad-CAM and attention-based methods, will be explored to improve interpretability and clinical trust. Furthermore, systematic hyperparameter optimization and lightweight model design will be investigated to enhance efficiency while maintaining high classification performance.

## 6 Conclusion

This study presents a novel framework for the automated classification of AML cells by integrating HCF with DL representations through the proposed HADNet feature fusion strategy and TCMA-Net classifier. The proposed approach effectively addresses key challenges such as class imbalance and the need for accurate and reliable diagnostic systems. Extensive experiments were conducted using multiple feature extraction strategies, including HCF, DenseNet201, MobileNetV2, and their hybrid combinations, evaluated under a consistent experimental setup. The results demonstrate that the proposed HADNet–TCMA-Net framework achieves superior performance compared to both traditional ML models and DL baselines, attaining a testing accuracy of 99.20% on the primary dataset. The effectiveness of the proposed method is further validated through ablation studies, which confirm the contribution of hybrid feature fusion in enhancing discriminative capability. Additionally, external validation on the ALL dataset demonstrates that the proposed framework maintains strong performance across different data distributions, confirming its robustness and generalization capability. Overall, the findings indicate that the integration of handcrafted and deep features significantly improves classification accuracy, stability, and reliability over conventional approaches. While the proposed framework demonstrates strong performance, future work will focus on extending evaluation to larger and more diverse datasets, incorporating advanced interpretability techniques, and optimizing model efficiency for real-world clinical deployment.

## Data Availability

Publicly available datasets were analyzed in this study. The dataset of AML Cytomorphology LMU has been taken from The Cancer Imaging Archive TCIA. The ALL dataset has been taken from kaggle https://www.kaggle.com/datasets/mehradaria/leukemia.
